# Comparative analysis of morphology and chloroplast genomes in endangered plant *Madhuca pasquieri* and two congeneric plants: revealing phylogenetic relationships

**DOI:** 10.3389/fpls.2026.1836586

**Published:** 2026-06-19

**Authors:** Ziyao Wang, Jingzhe Qiu, Jiayu Qin, Zengmao Lai, Zhengli Jiao, Yifei Ma, Jiaxin Liu, Lu Zhang

**Affiliations:** 1 College of Forestry and Landscape Architecture, South China Agricultural University, Guangzhou, China; 2 Yinzhan Forest Farm, Qingyuan, China; 3 School of Life Sciences, Guangzhou University, Guangzhou, China

**Keywords:** chloroplast genome, comparative analysis, *Madhuca*, *Madhuca pasquieri,*, molecular markers, phylogenetic tree

## Abstract

**Background:**

This study addresses the taxonomic ambiguity impeding the conservation of *Madhuca pasquieri* — an endangered tree species classified as vulnerable on the International Union for Conservation of Nature (IUCN) Red List and listed as a national second-class protected plant in China — by integrating morphological comparison with complete chloroplast genome analysis of three *Madhuca* species.

**Methods:**

We sequenced and assembled the complete chloroplast genomes of three *Madhuca* species from Illumina data. Genome annotation, codon usage, repeat elements, and comparative genomics analyses were performed using standard bioinformatic tools. Phylogenetic relationships were reconstructed with 69 orthologous genes using maximum likelihood.

**Results:**

Despite high morphological similarity among *M. pasquieri* and its congener *M. hainanensis* and *M. longifolia*, their highly conserved chloroplast genomes (159,613-159,726 bp, 131 genes) yielded critical molecular resources. We identified hypervariable intergenic spacers (e.g., trnK-atpH) and simple sequence repeats (SSRs) as candidate markers for species discrimination. Phylogenetic reconstruction strongly supported *M. pasquieri* and *M. hainanensis* as a sister clade, with *M. longifolia* as an earlier‑diverging lineage.

**Discussion/Conclusion:**

These findings offer a preliminary molecular framework to aid species identification and provide valuable genomic resources for the conservation of *M. pasquier*

## 1 Background

*Madhuca pasquieri* (Dubard) H. J. Lam, a large evergreen arbor, is a species of *Madhuca* in the Sapotaceae family, and has approximately 85 congeneric species worldwide. Only two of them are found in China, another species is *M. hainanensis* Chun et How. *M. pasquieri* is distributed mainly in northern Vietnam and southern China. In China, only Guangdong, Guangxi, and Yunnan Province have been reported (Kan et al., 2021). It has a dense crown, with a straight trunk, and the leaves contain latex. The seeds have an oil content of up to 30%, and the wood can be used as a medicine, and is suitable for extracting hard rubber and tannin extracts; thus, it is an excellent tree species for timber utilization, oil production, medicinal purposes, and landscaping (Hoang et al., 2015; Kan et al., 2020). However, owing to severe human-induced damage in the mid-to-late 20th century, combined with scarce understory seedlings and saplings, slow growth, and difficult natural regeneration, its wild distribution is now extremely rare. It is classified as vulnerable on the International Union for Conservation of Nature (IUCN) Red List, warning us that more attention should be given to saving it from extinction. However, only a limited number of studies have been conducted on *M. pasquieri*, and *M. pasquieri* and *M. hainanensis* exhibit high morphological similarity, making interspecific differentiation difficult at the visual level. Considering the high morphological convergence between the three species in this genus, genomic markers can provide preliminary phylogenetic insights to help clarify species boundaries, which is essential for informing the scientific conservation and sustainable utilization of this genus.

Advances in high-throughput sequencing have established that chloroplast (cp.) genome sequencing and is regarded as a pivotal tool in plant biology. Chloroplasts, semiautonomous organelles in green plants, harbor covalently closed circular DNA genomes with a conserved quadripartite structure consisting of a large single-copy (LSC) region, a small single-copy (SSC) region, and two inverted repeat (IR) regions (IRa and IRb). They are characterized by maternal inheritance and low recombination rate. Compared with nuclear genomes, cp. genomes exhibit moderate evolutionary rates in both coding and non-coding regions, making cp. genomes ideal for phylogenetic inference (Jansen et al., 2005). Reduced sequencing costs enable large-scale cp. genome acquisition, which outperforms traditional DNA barcoding in resolving taxonomic ambiguity. Applications range from species identification, such as super barcoding, to population genetics, illuminating plant speciation and evolution. With next-generation sequencing refinements, cp. genomics will further advance systematic botany and conservation genetics.

Additionally, as another *Madhuca* species distributed in China, although it is not native to China (Chen et al., 2025), *M. longifolia* shares morphological similarities with *M. pasquieri* and *M. hainanensis*. To further clarify the interspecific relationships and facilitate accurate species identification within *Madhuca*, *M. longifolia* is of significant research value in this area. Complete cp. genome characterization of *M. pasquieri*, *M. hainanensis* and *M. longifolia* has been reported (Daniell et al., 2016; He et al., 2024), yet no further genomic insights into *M. pasquieri* have been documented. In this investigation, comprehensive structural and genomic analyses were conducted, with comparative evaluations against related Sapotaceae taxa retrieved from GenBank. The objectives of this study were as follows (Kan et al., 2021): to characterize and compare three complete cp. genome structures of *M. pasquieri*, *M. hainanensis* and *M. longifolia* in detail (Kan et al., 2020); to provide further genomic information for better identification of *M. pasquieri*, and *M. hainanensis* and for a better understanding of the phylogeny of *Madhuca*.

## 2 Methods

### 2.1 Preparation of plant materials

For morphological analysis, fresh young leaf samples taken from six-year-old individuals and two-year-old seedlings of *M. pasquieri*, *M. hainanensis and M. longifolia* were collected from South China Agricultural University Arboretum, Guangdong Province, China (23°9’12.82” N, 113°21’35.70” E). At the time of observation, 10 healthy, similarly vigorous individuals per species were selected for detailed morphological characterization. Fresh young leaves were collected from each selected individual (six leaves per individual, grouped into three pairs as replicates) for comparative observation. All morphological descriptions were based on qualitative visual inspection; quantitative morphometric data were not recorded in this study. For cp. genome sequencing, three biological replicates were prepared for each species, with each replicate comprising three leaf blades. Voucher specimen: *M. pasquieri* (CANT MMP01), *M. hainanensis* (CANT MMH01), *M. longifolia* (CANT MMC01). Cultivated seedling, Tree nursery cultivation, South China Agricultural University, Guangzhou, China.

### 2.2 DNA extraction, genome sequencing, and annotation

We constructed a library with an average length of 350 bp via the Nextera XT DNA Libraries Preparation Kit (Illumina, San Diego, CA). The libraries were then sequenced on the Illumina NovaSeq 6000 platform. The raw sequence reads were subjected to quality control processing via Fastp (version 0.19.7). Following data processing, 5.09 Gb of raw sequencing data for *M. pasquier*i yielded 5.02 Gb of high-quality clean data (GC content: 35.82%; Q20: 98.43%; Q30: 95.79%). This resulted in a cp. genome coverage depth of 3,207.98×. For *M. hainanensis*, 7.78 Gb of raw data were processed to 7.71 Gb of clean data (GC content: 35.41%; Q20: 97.81%; Q30: 94.00%), achieving a coverage depth of 3,028.74× for the cp. genome. Similarly, *M. longifolia* generated 7.16 Gb of clean data from 7.24 Gb of raw data (GC content: 35.23%; Q20: 97.72%; Q30: 93.97%), providing a cp. genome coverage of 3,111.49×. These metrics collectively demonstrate the high quality and reliability of both the sequencing data and the assembled cp. genomes. The high-quality reads were assembled into the cp. genome using *de novo* assembler SPAdes v.3.14.1 software (Bankevich et al., 2012). Cp. genome annotation was performed using PGA (Qu et al., 2019) with default parameters, using *M. hainanensis* (GenBank: NC_053619) as the reference. Annotation thresholds included a sequence similarity of 40%, an e-value cutoff of 1e-5, and a minimum alignment length of 30 amino acids for protein-coding genes. Genes were identified based on homology to reference sequences; pseudogenes and redundant or low-quality gene models (short, incomplete, or abnormally long) were filtered out, and only intact, high-confidence genes were included in the final annotation.

### 2.3 Analyses of codon usage, repeat sequence and SSR

Protein-coding sequences were extracted from the cp. genomes via a Perl script. To ensure data reliability, sequences shorter than 300 bp and duplicate entries were removed. Only sequences initiating with an ATG start codon and terminating with a TAA, TAG, or TGA stop codon were selected for subsequent analysis. Codon usage bias was assessed using CodonW (version 1.4.2) software (Rice et al., 2000), which was also employed to calculate the RSCU values for the protein-coding genes.

Simple sequence repeat (SSR) loci were identified via MISA software (MicroSAtellite identification tool; https://webblast.ipk-gatersleben.de/misa/). The search was performed with the following parameters: a minimum of 10 repeats for mononucleotide SSRs, 5 repeats for dinucleotides, 4 repeats for trinucleotides, and 3 repeats for tetra-, penta-, and hexanucleotides (Beier et al., 2017).

Tandem repeats were predicted using Tandem Repeats Finder (TRF) with the following parameters: match = 2, mismatch = 7, indel = 7, match probability = 80, indel probability = 10, minimum alignment score = 50, maximum period size = 500 (Benson, 1999). Dispersed repeats were predicted via Vmatch (http://www.vmatch.de/) with the following parameter: -d -p -h 3 -l 30 -best 50 -noscore -noidentity -absolute (Kurtz, 2003).

### 2.4 Analysis of sequence divergence and nucleotide diversity

The structural dynamics at the junctions of the IR regions were assessed using the online tool IRscope (https://irscope.shinyapps.io/irapp/) to detect potential contraction and expansion events (Amiryousefi et al., 2018). Using the *M. pasquieri* cp. genome as a reference, global alignment of the cp. genome sequences of three *Madhuca* species were analyzed with MAFFT software (v7.429) (Katoh and Standley, 2013). Nucleotide diversity (Pi) across the complete cp. genomes were subsequently analyzed using a sliding window approach in DnaSP v6, with a window length of 600 bp and a step size of 200 bp (Rozas et al., 2017). The annotation file of the *M. pasquieri* cp. genome was converted into the format required by mVISTA using a custom Perl script. Subsequent analysis was performed with the online mVISTA platform (http://genome.lbl.gov/vista/mvista/submit.shtml) under the Shuffle-LAGAN mode, by submitting the cp. genome sequences and corresponding processed annotation files of *M. hainanensis* and *M. longifolia*. In the resulting plot, the vertical axis represents the percent identity (50%–100%) of each sample to the reference sequence (Brudno et al., 2003; Frazer et al., 2004).

### 2.5 Phylogenetic analysis

Orthologous single-copy genes were identified via OrthoFinder v2.3.14, yielding a total of 69 genes (*accD, atpA, atpB, atpE, atpF, atpH, atpI, ccsA, cemA, clpP, infA, matK, ndhA, ndhC, ndhD, ndhE, ndhF, ndhG, ndhH, ndhI, ndhJ, ndhK, petA, petB, petD, petG, petL, petN, psaC, psaI, psaJ, psbA, psbB, psbC, psbD, psbE, psbF, psbH, psbI, psbJ, psbK, psbL, psbM, psbN, psbT, psbZ, rbcL, rpl14, rpl16, rpl20, rpl22, rpl33, rpl36, rpoA, rpoB, rpoC1, rpoC2, rps11, rps14, rps15, rps16, rps18, rps19, rps2, rps3, rps4, rps8, ycf3, ycf4*). The protein-coding sequences of these 69 genes from 15 samples were individually aligned using MAFFT (v7.429). The resulting multiple sequence alignments were then refined with Gblocks 0.91 b to trim poorly aligned positions and regions containing gaps. The curated alignments for all genes were subsequently concatenated into a supermatrix for phylogenetic tree construction. *Amborella trichopoda* (NC 005086) was used as the outgroup. For maximum likelihood tree inference, IQ-TREE v1.6.1 was employed (Editoral Committee of Flora of China, Chinese Academy of Sciences, 1987). The best-fit nucleotide substitution model was selected automatically using the ModelFinder utility implemented in IQ-TREE, which is based on the Bayesian information criterion (Sankara Rao et al., 2016). The GTR+F+G4 model was identified as optimal. Branch support was assessed with 1000 bootstrap replicates.

## 3 Results

### 3.1 Morphological comparison

*M. pasquieri* is an evergreen tree reaching up to 30 m in height, with a diameter at breast height (DBH) of up to 60 cm. The bark is gray−black and exudes yellowish−white latex. Young branches are initially rusty−tomentose, becoming glabrescent. Leaves are alternate, clustered or scattered at branch tips, coriaceous, obovate to obovate−oblong, measuring 6–16 × 2–6 cm. The leaf apex is broadly acute to obtuse, the base cuneate, and the margin revolute. Lateral veins number 13–22 (–26) pairs. The adaxial leaf surface is glabrous, sometimes glossy. Flowers are borne in several−flowered axillary fascicles; pedicels are 1.5–3.5 cm long, rusty−tomentose. The calyx has 4 (–5) ovate lobes, 3–6 mm long, tomentose. The corolla is yellowish−green with 6–11 lobes. Fertile stamens number (16–)18–22 (–24). The ovary is 6–8−locular. The fruit is ellipsoid to subglobose, 2–3 × 1.5–2 cm. Each fruit contains 1–5 ellipsoid seeds, 1.8–2.7 × 1–1.2 cm. Endosperm is absent, and the cotyledons are flat and oily (Čorak et al., 2022).

*M. hainanensis* is an evergreen tree 9–30 m tall. The bark is dark gray−brown and exudes yellowish−white sticky latex. Young branches are densely rusty−red villous. Leaves are clustered at branch tips, coriaceous, oblong−obovate to oblong−oblanceolate, measuring 6–12 × 2.5–4 cm. The leaf apex is rounded to retuse, the base attenuate and decurrent. Lateral veins are very fine, numbering 20–30 pairs. The adaxial leaf surface is glossy and glabrous. Flowers are arranged in 1–3−flowered axillary cymes; pedicels are 2–3 cm long, densely rusty−sericeous. The calyx has four lobes, the outer two larger (1.5–8 (–12) mm) and the inner two smaller, all densely rusty−tomentose. The corolla is white with 8–10 lobes. Fertile stamens number 28–30, arranged in three whorls. The ovary is 6–8−locular. The fruit is greenish−yellow, ovoid to subglobose, 2.5–3 × 2–2.8 cm, pubescent. Each fruit contains 1–5 oblong−elliptic seeds, laterally compressed, 2–2.5 × 0.8–1.2 cm, glossy brown. Endosperm is absent (Čorak et al., 2022).

*M. longifolia* is a deciduous or semi−evergreen tree 15–20 m tall, with a DBH reaching up to 80 cm. The bark is gray to dark gray, longitudinally fissured and flaking, and exudes white latex. Leaves are alternate, clustered at branch tips, coriaceous, narrowly oblong to obovate or elliptic−lanceolate, measuring 7.5–23 × 4–12 cm. The leaf apex is acute to acuminate, the base cuneate, and the margin entire. Lateral veins number 10–15 pairs. The adaxial leaf surface is glabrous at maturity; young leaves are pinkish to reddish−brown and abaxially densely pubescent. Flowers are solitary or in 3–5−flowered axillary fascicles; pedicels are 2–5 cm long. The calyx has four ovate lobes, approximately 8 × 6 mm, the outer lobes nearly glabrous and the inner lobes slightly pubescent. The corolla is fleshy, yellowish−white to pale yellow−green, with 8–12 lobes. Fertile stamens number 16–24, arranged in two whorls, the upper whorl sessile and pubescent. The fruit is a berry, ellipsoid to ovoid, 2.5–5 cm long, yellow−green, fleshy. Each fruit contains 1–4 long−ellipsoid seeds, compressed, 2–5 × 1–1.2 cm, brown, glossy, with short beaks at both ends. Endosperm is absent (Kalyaanamoorthy et al., 2017).

**Figure 1:**
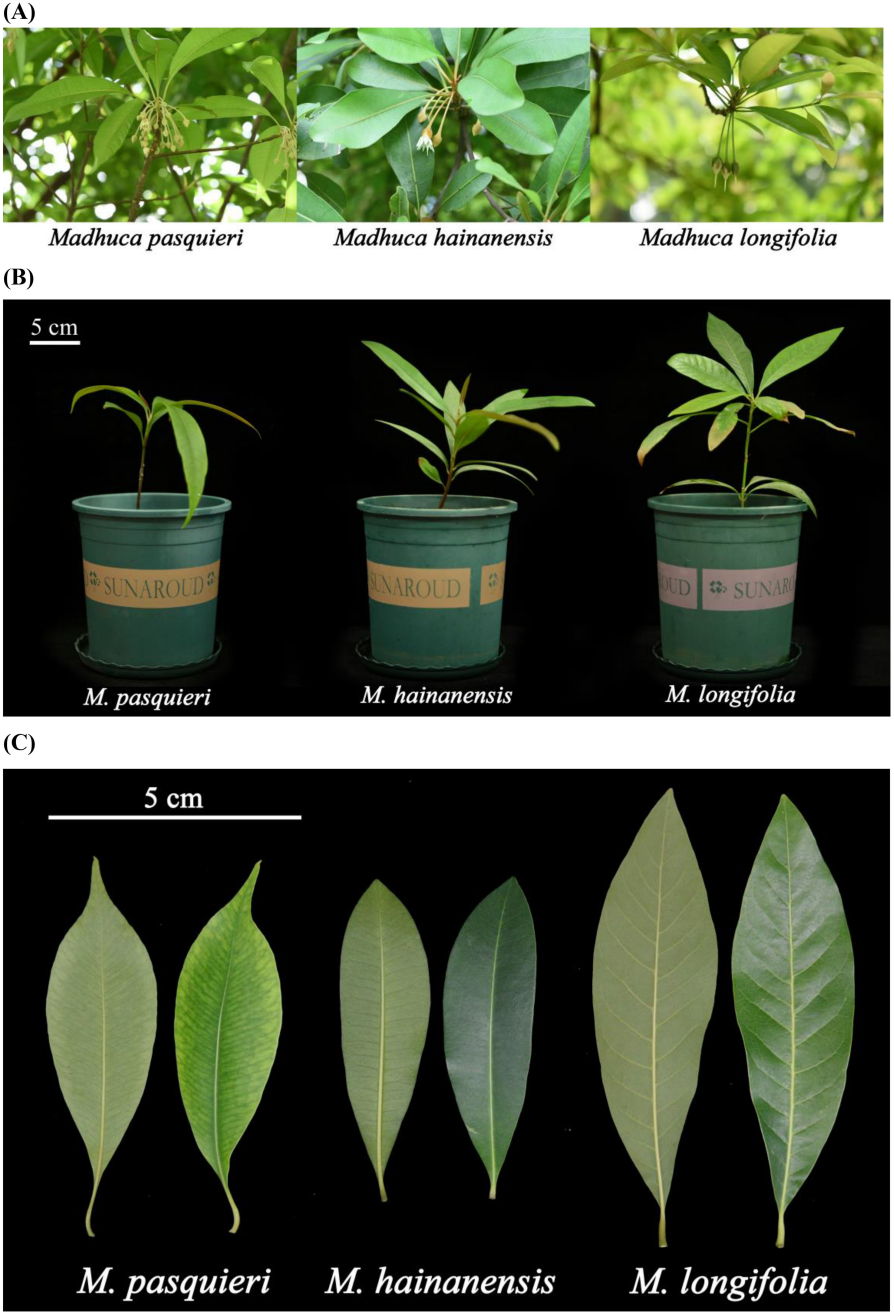
Morphological comparison of the three species of *Madhuca*. **(A)** flowers; **(B)** two-year-old seedlings; **(C)** fresh leaves.

A comparative morphology study of flowers, two-year-old seedlings, and leaves was conducted to delineate *M. pasquieri*, *M. hainanensis* and *M. longifolia*. All of these species produce similar pendulous axillary cymose inflorescences, with solitary flowers rarely occurring in *M. hainanensis* (**Figure 1A**). Our observations revealed similar floral morphologies between the two native endangered species, *M. pasquieri* and *M. hainanensis*, whereas the exotic *M. longifolia* was distinct. The calyx of *M. longifolia* is green and its petals are pale yellow-green, whereas the other two species have light brown calyces and white to yellowish-white petals. Furthermore, *M. hainanensis* can be distinguished from *M. pasquieri* by its slightly greater number of corolla lobes, although this minor difference poses a challenge to reliable visual identification.

Morphological comparison of two−year−old seedlings of the three species (**Figure 1B**) revealed that seedlings of *M. pasquieri* and *M. hainanensis* were visibly smaller and produced fewer leaves than those of *M. longifolia* over the same growth period. *M. pasquieri* seedlings also appeared less robust than those of the other two species. Morphologically, *M. pasquieri* produces fewer leaves than the other two species do over the same period, which may indicate that its seedling survival ability is significantly lower than that of the other two species. Similarly, the seedling survival ability of *M. hainanensis* was correspondingly lower than that of *M. longifolia*.

The foliar morphology of the three studied *Madhuca* species is characterized by alternate, simple leaves with entire, non-lobed margins. The leaves are coriaceous in texture and mostly obovate or obovate-oblong, with cuneate bases. The species differ in leaf thickness and margin: *M. pasquieri* and *M. hainanensis* have thicker leaves with revolute margins, whereas those of *M. longifolia* are more pliable and lie completely flat (**Figure 1C**). The three *Madhuca* species can be distinguished by subtle differences in leaf morphology, with the leaf apex serving as a primary diagnostic characteristic (Yano and Terashima, 2001). *M. pasquieri* exhibits a gradually broadened, blunt apex, while *M. hainanensis* is characterized by a conspicuously rounded and blunt apex. In contrast, the apex of *M. longifolia* is shortly acuminate. Leaf venation serves as a critical trait for distinguishing among these *Madhuca* species. *M. pasquieri* is characterized by a rounded, abaxially prominent midrib and lateral veins that are subtle adaxially but distinct abaxially. *M. hainanensis* displays a prominently raised abaxial midrib and relatively obvious, dense adaxial lateral veins. Conversely, *M. longifolia* is distinguished by a slightly raised abaxial midrib, yet it possesses the most conspicuous and relatively dispersed lateral veins, a key diagnostic characteristic for this species.

The accurate morphological identification of *Madhuca* species is often complicated by which arise from environmental heterogeneity and may reflect convergent evolution or phenotypic plasticity (Baumgartner et al., 2020), thereby impeding the development and implementation of effective conservation strategies for *M. pasquieri*. Consequently, integrating molecular tools is necessary to provide a preliminary phylogenetic framework for species delimitation, analyze population genetic structure, and inform conservation efforts for *M. pasquieri*, although robust species delimitation will ultimately require the integration of nuclear genomic data.

### 3.2 The general features of cp. genomes

**Figure 2:**
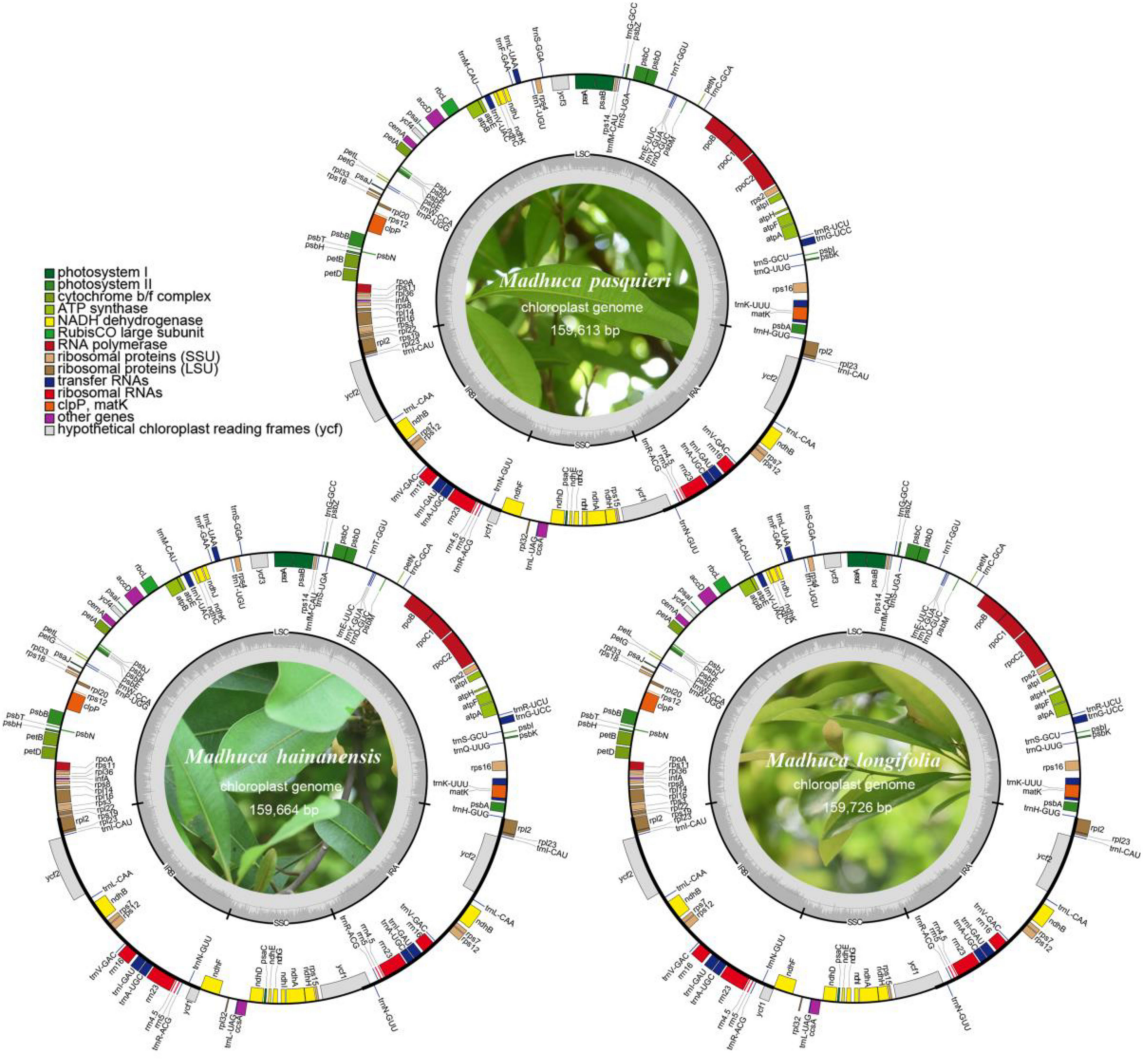
cp. genome map of three *Madhuca* species. Genes drawn inside the circle are transcribed clockwise, and genes outside are transcribed counterclockwise. Genes that possess different functions are color coded in the legend. The area in darker gray and lighter gray in the inner circle indicate the GC content and AT content, respectively. (cp., chloroplast).

The cp. genomes of the three *Madhuca* species are structurally conserved, with genome sizes ranging from 159,613 bp (*M. pasquieri*) to 159,726 bp (*M. longifolia*) (**Figure 2**). All three genomes possess a typical vascular plant quadripartite structure, which consists of two inverted repeats (IR) regions (26,087–26,093 bp), a large single-copy region (LSC, 88,828–88,958 bp), and a small single-copy region (SSC, 18,594–18,607 bp) (**Table 1**).

**Table 1:** Comparison of the genome characteristic of the three *Madhuca* chloroplasts.

Species	Size	Genome size(bp)			Number of genes				GC(%)
		LSC	SSC	IR	PCG	tRNA	rRNA	Total	
*Madhuca pasquieri*	159613	88828	18607	26089	86	37	8	131	36.80
*Madhuca hainanensis*	159664	88876	18602	26093	86	37	8	131	36.79
*Madhuca longifolia*	159726	88958	18594	26087	86	37	8	131	36.75

LSC, large single copy; SSC, small single copy; IR, inverted repeat; PCG, protein coding genes.

Among the three cp. genomes, the GC contents of the LSC, SSC, and IR regions were 36.8%, 36.79%, and 36.75%, respectively. Their cp. genomes all contained 131 genes, including 86 known protein-coding genes (PCGs), 37 tRNA genes and 8 rRNA genes. The three *Madhuca* species were completely identical in their gene in functional classification, including 46 photosynthesis related genes (e.g. *rbcL*, *ndhA, atpA*, etc.), 74 transcription and translation related genes (e.g. *rps11*, *rpl14*, *rpoA*, etc.), 6 other genes (e.g. *matK*, *ccsA*, *cemA*, etc.), and 5 unknown function gene (*ycf1, ycf2, ycf4*), including 15 genes with one intron (e.g. *ndhA*, *ndhB*, *petB*, etc.) and 2 genes with two introns (*ycf3*, *clpP*). Additionally, the rps12 gene was trans-spliced (**Table 2**).

**Table 2:** Functional classification of the chloroplast genes of three samples from *Madhuca*.

Classification	Group of genes	Gene name
Photosynthesis related genes	Subunits of NADH-dehydrogenase	ndhA*, ndhB* (×2)*, ndhC, ndhD, ndhE, ndhF, ndhG, ndhH, ndhI, ndhJ, ndhK*
	Subunits of photosystem I	psaA, psaB, psaC, psaI, psaJ, ycf3**
	Subunits of photosystem II	*psbA, psbB, psbC, psbD, psbE, psbF, psbH, psbI, psbJ, psbK, psbL, psbM, psbN, psbT, psbZ*
	Subunits of cytochrome b/f complex	petA, petB*, petD*, petG, petL, petN
	Subunits of ATP synthase	atpA, atpB, atpE, atpF*, atpH, atpI
	Subunits of Large subunit of rubisco	*rbcL*
Transcription and translation related genes	Small subunit of ribosome	rps11, rps12*(×2), rps14, rps15, rps16*, rps18, rps19, rps2, rps3, rps4, rps7(×2)*, rps8*
	Large subunit of ribosome	rpl14, rpl16*, rpl2* (×2), *rpl20, rpl22, rpl23*(×2)*, rpl32, rpl33, rpl36*
	DNA dependent RNA polymerase	rpoA, rpoB, rpoC1*, rpoC2
	rRNA genes	*rrn16*(×2)*, rrn23*(×2)*, rrn4.5*(×2)*, rrn5*(×2)
	tRNA genes	trnA-UGC* (×2), trnC-GCA, trnD-GUC, trnE-UUC, trnF-GAA, trnG-GCC, trnG-UCC*, trnH-GUG, trnI-CAU(×2), trnI-GAU* (×2), trnK-UUU*, trnL-CAA(×2), trnL-UAA*, trnL-UAG, trnM-CAU, trnN-GUU(×2)*, trnP-UGG, trnQ-UUG, trnR-ACG*(×2)*, trnR-UCU, trnS-GCU, trnS-GGA, trnS-UGA, trnT-GGU, trnT-UGU, trnV-GAC*(×2), trnV-UAC*, trnW-CCA, trnY-GUA, trnfM-CAU
Others genes	Maturase	*matK*
	c-type cytochrom synthesis gene	*ccsA*
	Envelope membrane protein	*cemA*
	Protease	clpP**
	Subunit of Acetyl-CoA-carboxylase	*accD*
	Translation initiation factor IF-1	*infA*
Unknown function genes	Genes of unknown functions Open Reading	*ycf1*(×2)*, ycf2*(×2)*, ycf4*

Gene*: Gene with one intron; Gene**: Gene with two introns; Gene (×2): Genes duplicated in the IR regions; Gene^#^: Gene was trans-spliced.

### 3.3 Codon usage analysis

**Figure 3:**
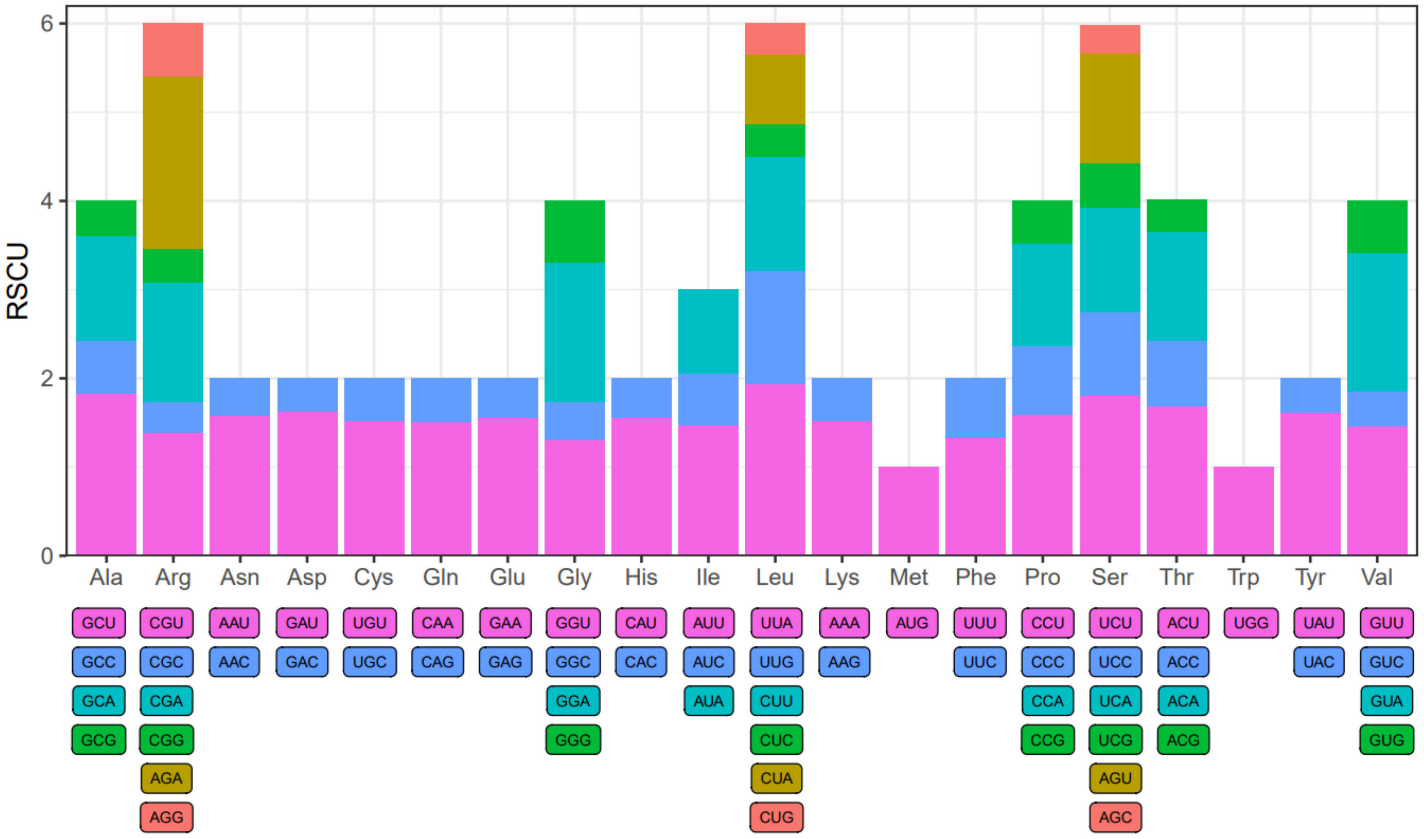
Codon contents of the CDS sequences of the three *Madhuca* species cp. genomes. The ordinate shows the RSCU values, and the abscissa represents 20 amino acids. The histogram represents codon usage for each amino acid of the three species. (CDS, Coding sequences; RSCU, Relative synonymous codon usage).

The codon usage in the cp. genomes of the three species was highly similar. The codon usage of protein-coding genes in the cp. genomes of the three *Madhuca* species was examined. The concatenated coding sequences comprised a total of 20,899 codons (including the three stop codons TAA, TAG, and TGA) and encoded 20 amino acids. Relative synonymous codon usage (RSCU) was computed for the three cp. genomes as well. The RSCU values of three cp. genomes showed highly similarity, with six codons for leucine (Leu), arginine (Arg), and serine (Ser); four codons for alanine (Ala), glycine (Gly), proline (Pro), and threonine (Thr); and three codons for isoleucine (Ile). Apart from methionine (Met) and tryptophan (Trp), which are each encoded by a single codon, the other nine amino acids have two codons each (**Figure 3**). In *M. pasquieri* and *M. longifolia*, leucine (Leu: 2,165 occurrences, 10.36%) was the most abundant amino acid, whereas cysteine (Cys: 225, 1.08%) was the least frequent. *M. hainanensis* is largely comparable to the other two species, although minor variations mainly in the numbers of certain codons were observed. The codon with the highest RSCU value was GAA (1.55), encoding glutamate (Glu), followed by AUU (1.47), encoding isoleucine (Ile) (**Additional File 1-3: Supplementary Table 1**–**3**).

### 3.4 Long repeat and SSR analyses

Long tandem repeats are defined as repeat motifs longer than 6 bp, and dispersed repeats are defined as dispersed sequences of at least 30 bp. These structural features contribute significantly to cp. genome rearrangement and promote genetic diversity within populations (Nie et al., 2012). In contrast, simple sequence repeats (SSRs), also termed DNA microsatellites, consist of short tandemly repeated units of 1–6 bp that are widely distributed across the cp. genome (Li et al., 2002).

**Figure 4:**
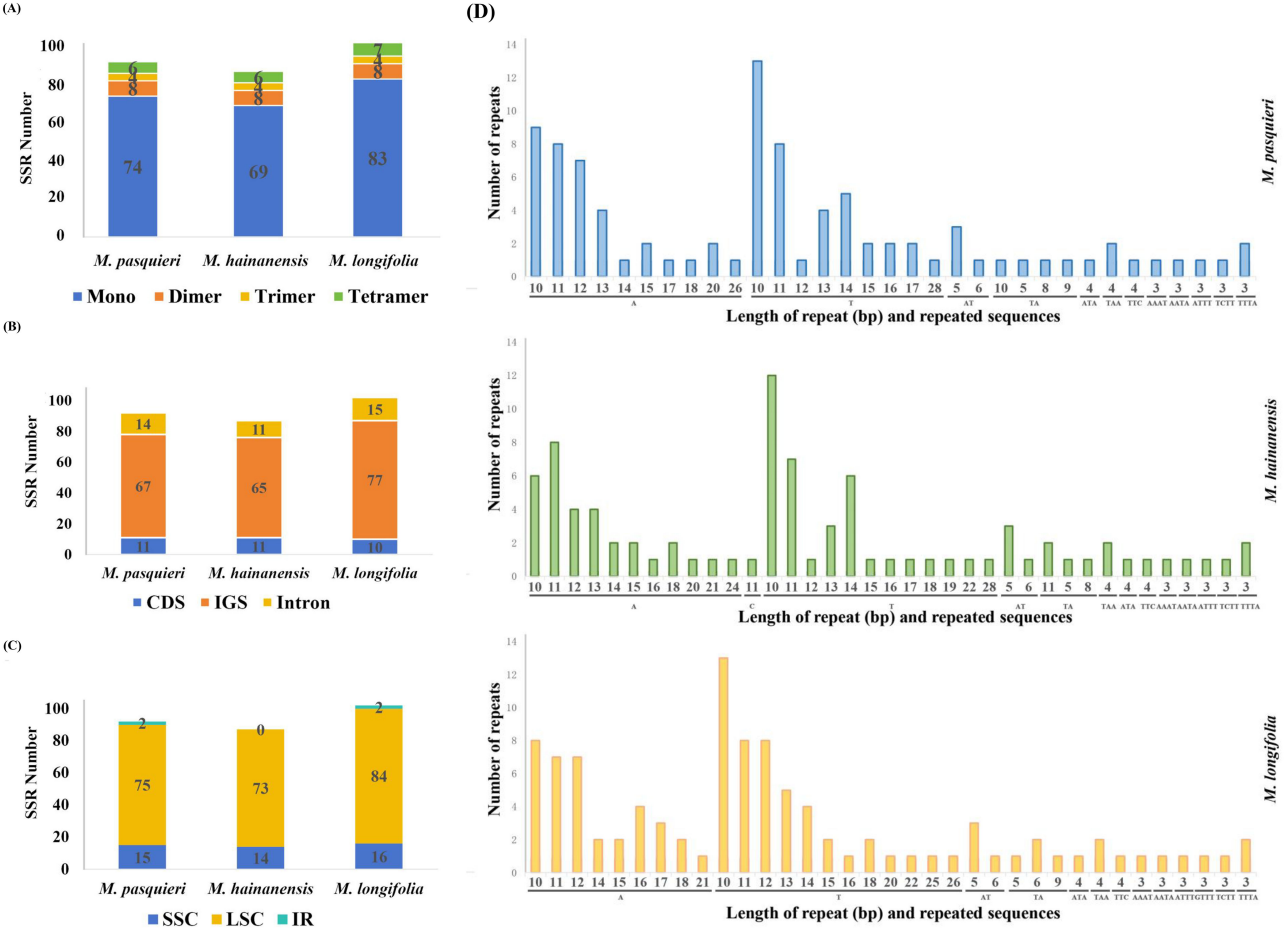
Comparative analyses of repeat sequences. **(A)** Types and numbers of SSRs; **(B)** Frequencies of identified SSRs in CDS, IGS, and intron; **(C)** Frequencies of identified SSRs in the LSC, SSC, and IR regions; **(D)** SSR types in the cp. genomes of the three *Madhuca* species. (SSR, Simple sequence repeat; CDS, Coding sequences; IGS, Intergenic spacers; LSC, Large single-copy; SSC, Small single-copy; IR, Inverted repeat).

In the cp. genomes of *M. pasquieri*, *M. hainanensis*, and *M. longifolia*, we identified 92, 87, and 102 SSRs, respectively. Mononucleotide repeats were the most prevalent type, followed by dinucleotide repeats, with trinucleotide repeats being the least common (**Figure 4A**). Over 70% of these SSRs were located in intergenic spacer (IGS) and LSC region (**Figures 4B, C**). Additionally, poly(A) and poly(T) motifs constituted more than 80% of the SSRs in *M. pasquieri* and *M. longifolia*, and 78.2% of those in *M. hainanensis*, reflecting a pronounced A/T bias in the SSR compositions of these three species (**Figure 4D**).

**Figure 5:**
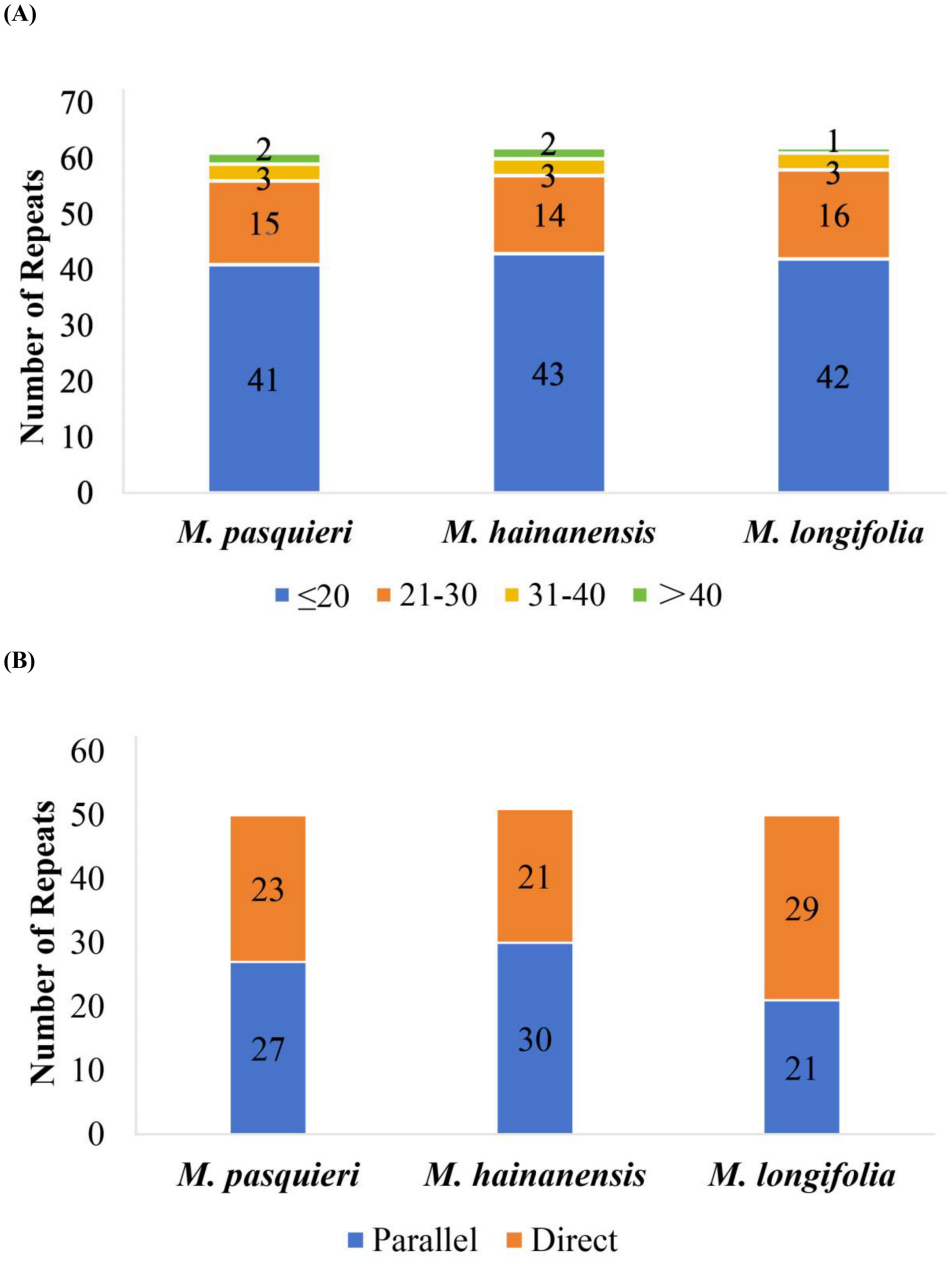
Numbers of long repeats. **(A)** Number of tandem repeats; **(B)** Number of dispersed repeats.

Long repeat sequences, including both tandem and dispersed repeats, were identified in the three *Madhuca* species (**Figure 5**). A total of 62 tandem repeats were identified in both *M. hainanensis* and *M. longifolia*, while 61 were found in *M. pasquieri*. The lengths of these tandem repeats were predominantly shorter than 40 bp, with most being under 20 bp. Two major types of dispersed repeats were also identified, with the total counts varying slightly among the species (**Figure 5A**, **Additional File 4-6**: **Supplementary Table 4**–**6**). Notably, both *M. pasquieri* and *M. hainanensis* showed a lower ratio of parallel to direct repeats than did *M. longifolia*. Furthermore, *M. hainanensis* exhibited the greatest abundance of dispersed repeats, with a total of 51 repeats, whereas 50 repeats were identified in both *M. pasquieri* and *M. longifolia* (**Figure 5B**).

### 3.5 IR contraction and expansion

**Figure 6:**
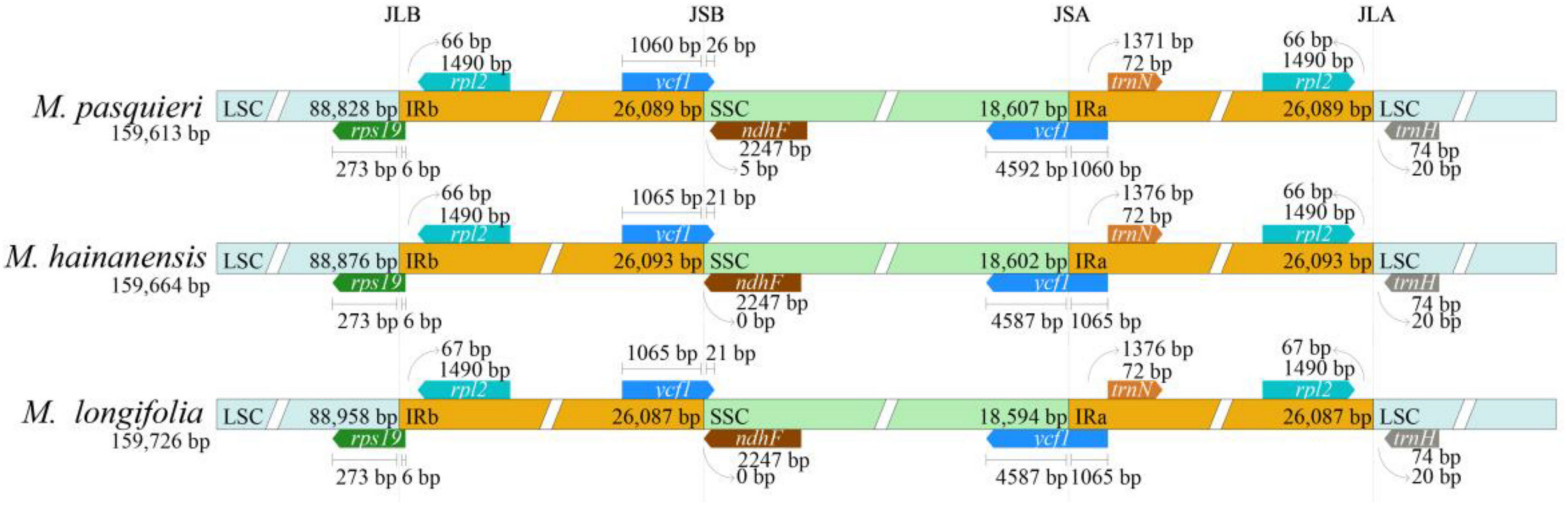
Comparison of the LSC, IR, and SSC junctions. The colored boxes for genes represent the gene position. JLB denotes the LSC/IRb junction, JSB denotes the SSC/IRb junction, JSA denotes the SSC/IRa junction, and JLA denotes the LSC/IRa junction. (LSC, Large single-copy; SSC, Small single-copy; IR, Inverted repeat).

Comparative sequence analysis between the three *Madhuca* species revealed that the cp. genomes were highly conserved in their structure, gene number, and gene sequence (**Figure 6**). However, there were still some structural variations in the junctions. The LSC/IRa junction was located between the *rpl2* and *trnH* genes. The *trnH* gene is located in the LSC region, as observed in some dicots (Asano et al., 2004; Souza et al., 2019). The LSC/IRb junction was located within the *rps19* gene, with the IRb-located *rpl2* gene was 66 bp from the LSC region, whereas in *M. longifolia* the corresponding distance was 67 bp. The SSC/IRa junction lies within the *ycf1* gene, and the *ycf1* gene in the IRa region extends into the SSC region by 4592 bp in *M. pasquieri*, whereas it extends 4587 bp in the other two species. The IRb copy of *ycf1*, with a length of 1086 bp in all species, extends into the SSC region by 21 bp in *M. hainanensis* and *M. longifolia*, and 26 bp in *M. pasquieri*, resulting in the duplication of the *ycf1* pseudogene in the IRb region. Furthermore, at the LSC/IRa junction, the *rpl2* gene in *M. longifolia* is situated 1 bp farther from the LSC region than its two congeners.

### 3.6 Comparative genomic divergence and genome rearrangement

**Figure 7:**
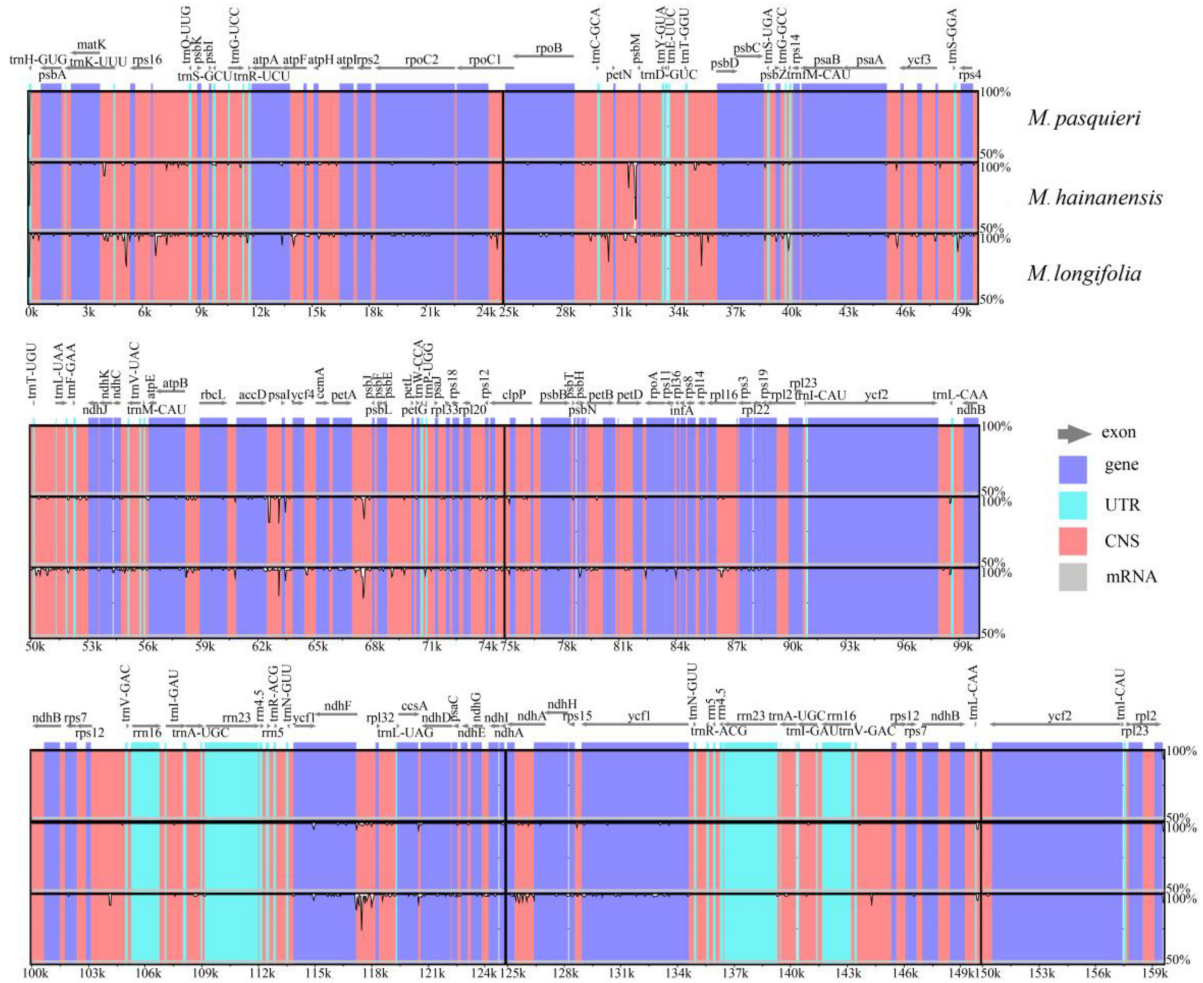
Global alignment of three *Madhuca* cp. genomes using the annotation of *M. pasquieri* as a reference. The dark blue blocks indicate exons, and the red blocks indicate conserved non-coding sequences. tRNA and rRNA genes are denoted by cyan blocks. The white peaks represent regions with sequence variation among the cp. genomes. Arrows are drawn above the graph indicating the direction of genes. The vertical axis indicates sequence identity between 50% and 100%.

To further investigate sequence divergence at the chloroplast genomic level among *Madhuca* species, we performed a genome-wide alignment to analyze polymorphic regions. The LSC and SSC regions showed substantial sequence variation, while the IR regions were the most conserved. Most variable sites were located in non-coding regions, where the degree of variation was significantly greater than that in coding regions. The mVISTA analysis further identified four highly divergent regions located in the intergenic spacers between *trnK*-*atpH*, *rpoB*-*rps14*, *accD*-*psbJ*, and *ndhF*-*ndhH* (**Figure 7**). These results indicate that these regions are divergent among the three examined *Madhuca* species.

**Figure 8:**
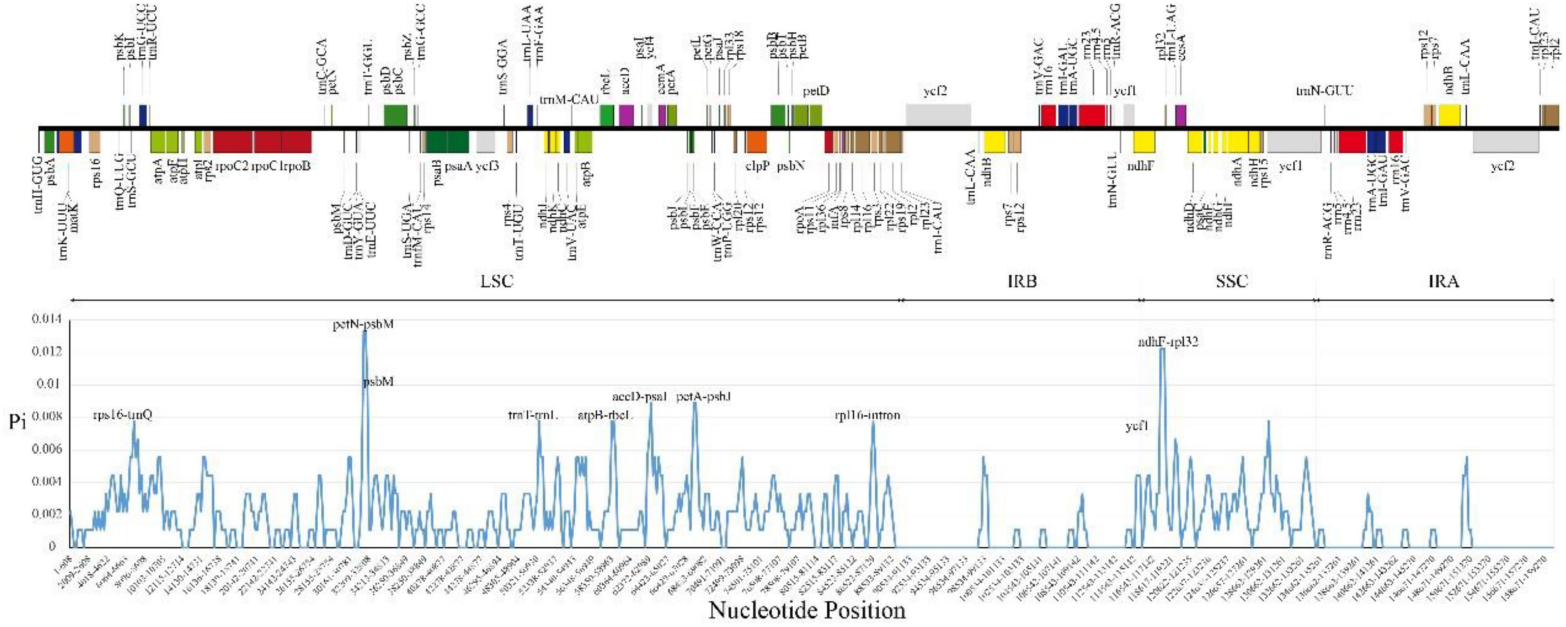
Sliding window analysis of the complete cp. genomes of three *Madhuca* species. The X-axis indicates the midpoint of the window. The Y-axis indicates the nucleotide diversity within each window. (Pi: Nucleotide diversity).

Nucleotide diversity (Pi) analysis was further employed to identify hypervariable regions. Sliding-window analysis of sequence polymorphisms across the three *Madhuca* cp. genomes revealed an average Pi value of 0.00169. The IR regions displayed lower nucleotide diversity than the single-copy regions, while the Pi values of non-coding regions were significantly higher than those of the coding regions. Furthermore, ten non-coding segments in the LSC/SSC regions with relatively high Pi values were identified as hypervariable regions (**Figure 8**). These included seven IGS regions (*rps16*-*trnQ*, *petN*-*psbM*, *trnT*-*trnL*, *atpB*-*rbcL*, *accD*-*psaI*, *petA*-*psbJ*, and *ndhF*-*rpl32*), two protein-coding regions (*psbM* and *ycf1*), and one intron (*rpl16*). These findings are consistent with the results from the mVISTA analysis.

### 3.7 Phylogenetic relationships

**Figure 9:**
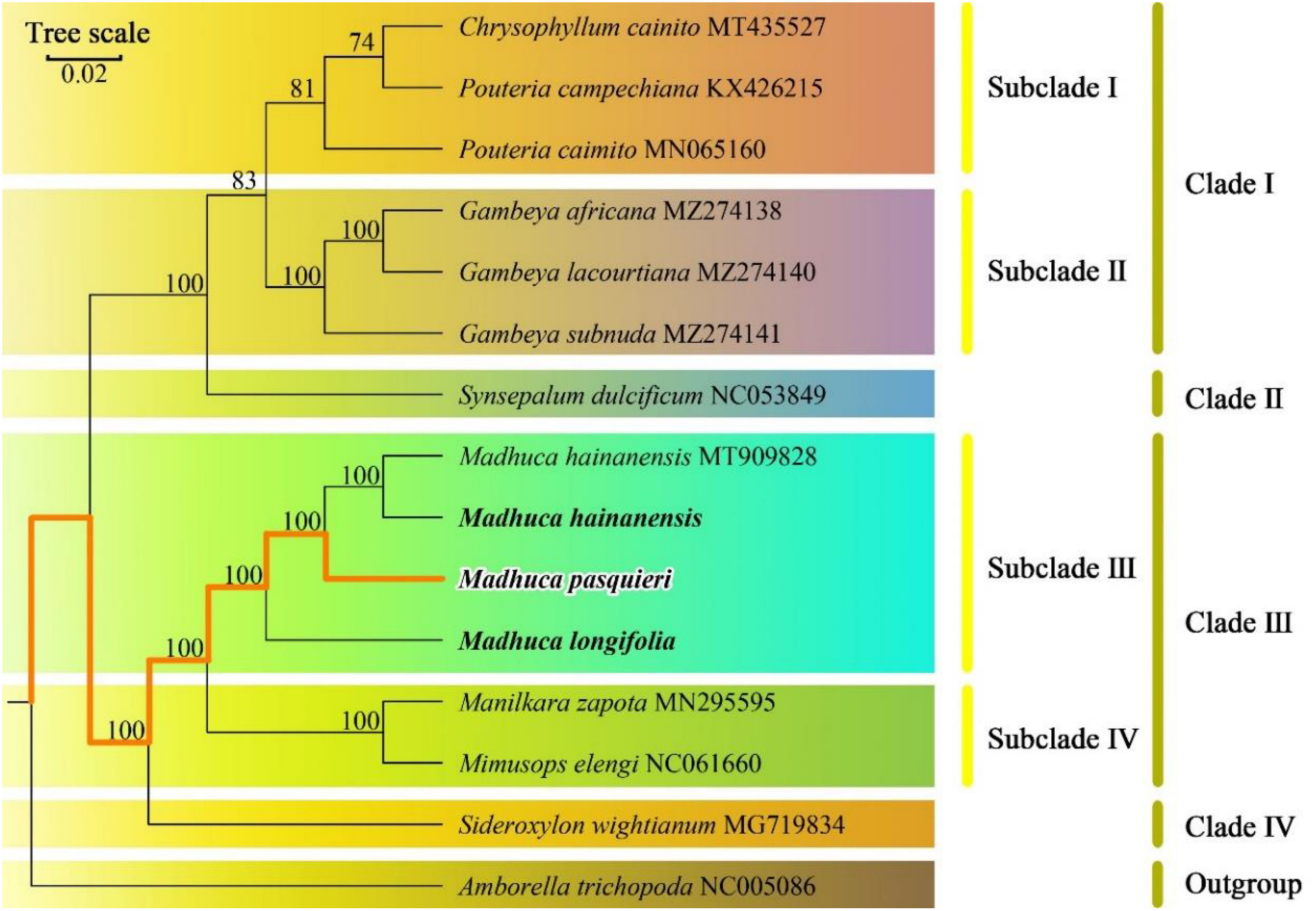
Phylogenetic tree generated via maximum likelihood analysis on the basis of complete cp. genomes. (cp., chloroplast).

To explore the interspecific relationships of the three samples and the phylogenetic relationships of Sapotaceae species, the cp. genomes of three newly sequenced species, along with the 11 complete cp. genome sequences of Sapotaceae were downloaded from GenBank (with *Amborella trichopoda* as the outgroup), and used to construct the Sapotaceae phylogeny (Additional file 7: **Supplementary Table 7**). A phylogenetic tree was constructed using maximum likelihood analysis. The phylogenetic tree (**Figure 9**) contained 12 nodes. The majority of these nodes were well-supported, with bootstrap values exceeding 81%. A single node received support below this threshold, whereas nine nodes were strongly supported at 100%, collectively indicating robust phylogenetic reconstruction. As shown, 13 Sapotaceae species were clustered into four clades, and *Amborella trichopoda* formed a single branch. Clade I included *Chrysophyllum cainito*, *Pouteria campechiana*, *P. caimito*, and three *Gambeya* species. Within this clade, the three *Gambeya* species formed a distinct subclade separate from the cluster of the other three taxa. *Synsepalum dulcificum* formed an independent lineage. Clade III included *Manilkara zapota*, *Mimusops elengi*, and the three *Madhuca* species, with the *Madhuca* species forming a subclade distinct from that containing *Manilkara zapota* and *Mimusops elengi*. *Sideroxylon wightianum* also occupied an isolated position. These relationships demonstrate that *C. cainito*, *P. campechiana*, and *P. caimito* share closer phylogenetic affinities with each other than with the *Gambeya* species. Together with *Synsepalum. dulcificum*, these six taxa constitute a major lineage that is phylogenetically distant from *Madhuca* within the family. In contrast, the three *Madhuca* species are closely related to *Manilkara zapota*, *Mimusops elengi*, and *S. wightianum*, among which *Manilkara zapota* and *Mimusops elengi* are the closest relatives. Within the genus *Madhuca*, *M. pasquieri* and *M. hainanensis* are more closely related, while *M. longifolia* represents an earlier-diverging lineage.

## 4 Discussion

### 4.1 Morphological similarity and challenges for identification

Comparative morphological analysis revealed that the leaves and flowers of *M. pasquieri* are difficult to distinguish from those of *M. hainanensis*. Owing to the significantly slower growth rates of their seedlings, visual identification of these two species is particularly challenging. Furthermore, field investigations revealed considerable variation in leaf morphology among *M. pasquieri* individuals across different habitats. This likely indicates strong phenotypic plasticity in *M. pasquieri*, potentially driven by factors such as the light conditions and topographic features (e.g., slope aspect and gradient) in its native habitat (Freschet et al., 2018; Chen et al., 2022). These factors collectively rendering morphology-based identification unreliable, necessitating more in-depth molecular analyses.

### 4.2 Cp. genome structure and comparative genomics

The cp. genomes of the three *Madhuca* species consist of an LSC region, an SSC region, and two IR regions, which exhibit a typical quadripartite structure consistent with other reported members of Sapotaceae (He et al., 2024), indicating high structural stability. A total of 131 genes were annotated in all three species, conforming to the basic pattern of angiosperm cp. genomes (Pan et al., 2026). Multiple sequence alignment of the three cp. genomes revealed high similarity in both genome architecture and sequence composition (**Figure 7**). As a universal feature of cp. genomes, single-copy regions exhibit greater variation, IR regions are the most conserved, and sequence divergence is higher in non-coding regions than in coding regions (Li et al., 2016).

Nucleotide diversity (Pi) is an indicator of DNA sequence variation, reflecting the level of genetic diversity within a species (Akhunov et al., 2010). According to the rule of thumb, the critical value for nucleotide diversity is 0.005, with higher values indicating greater population diversity. In interspecific comparisons of closely related species, the Pi values are generally less than 0.007 (Xiao et al., 2024; Tao et al., 2025). With the *M. pasquieri* cp. genome used as a reference, the calculated Pi value was 0.00169 (**Figure 8**), indicating a low degree of differentiation among the three species.

Codon usage patterns in cp. genomes are generally conserved across plants and are influenced by the combined effects of mutation pressure and natural selection (Wang et al., 2023). The codon usage in the three *Madhuca* cp. genomes exhibits a marked translational adaptation, characterized by a predominant selection of A- and T-ending synonymous codons. The genomic basis for this adaptation is rooted in a pronounced mutational bias toward A/T nucleotides, a hallmark of chloroplast DNA evolution (Bu et al., 2021). This intrinsic bias establishes the foundation for codon choice, upon which natural selection may act to fine-tune translational speed and accuracy, ultimately linking codon usage patterns to gene expression levels and functional constraints (Tatarinova et al., 2010; Im and Choi, 2017). An RSCU value greater than 1.0 indicates that the codon is used more frequently than expected by random chance, indicating a codon usage preference that is often correlated with tRNA abundance and translation efficiency (Wang et al., 2020).

SSRs, characterized by their polymorphic, abundance, and stability in eukaryotic genomes (Powell et al., 1995; Yuan et al., 2010), serve as effective molecular markers because their distinct compositional profiles across plant species facilitate their applications in taxonomy and diversity studies (Du et al., 2012; Chmielewski et al., 2015). We detected 92, 87, and 102 SSR loci in the cp. genomes of *M. pasquieri*, *M. hainanensis*, and *M. longifolia*, respectively. Mononucleotide repeats were the most abundant, followed by dinucleotide repeats. These two types primarily consisted of A/T and AT/TA motifs. The remaining SSRs were also predominantly composed of A and T bases, which is consistent with the observations from the codon usage bias analysis. This finding supports the view that SSRs in cp. genomes exhibit a significant AT bias, potentially because A-T base pairs form two hydrogen bonds, while G-C pairs form three, making AT-rich regions more prone to denaturation (Hu et al., 2024).

The repositioning of IR/SC borders constitutes a core mechanism in cp. genome evolution, serving as a primary source of architectural variation (Lilly et al., 2001; Li et al., 2021; Zhang et al., 2025). These boundary shifts, which have been extensively documented in recent angiosperm studies, often involve genes such as *ycf1* and generate lineage-specific structural fingerprints. Consequently, they serve as critical markers for deciphering phylogenetic relationships and understanding the dynamics of genomic diversification within plant families. Analysis of the IR/SC boundaries in the three *Madhuca* species revealed that the *ycf1* gene is located at the SSC/IRa junction in all three cp. genomes, leading to its pseudogenization—a sign of conserved IR boundaries (Yu et al., 2017; Abdullah et al., 2020; Abdullah et al., 2021). However, unlike its two congeners, *M. pasquieri* shows a 5 bp shift in *ycf1*, indicating a unique, minor expansion or contraction event in its evolutionary lineage. Furthermore, the mVISTA analysis pinpointed several highly variable intergenic spacers (e.g., *trnK*-*atpH*, *rpoB*-*rps14*, *accD*-*psbJ*, and *ndhF*-*ndhH*), which represent promising candidate regions for the development of specific molecular markers (**Figure 7**).

### 4.3 Phylogenetic relationships within *Madhuca*

Phylogenetic analysis on the basis of complete cp. genomes reconstructed the relationships among select Sapotaceae genera with exceptionally high support (He et al., 2024). This study clarified that the genus *Madhuca* forms a distinct evolutionary clade with *Manilkara* and *Mimusops*, which is clearly differentiated from other lineages within the family, such as the *Chrysophyllum-Pouteria* clade—a finding that is consistent with previous research. Within the genus, *M. pasquieri* and *M. hainanensis* are confirmed to be a pair of recently diverged sister species, while *M. longifolia* represents an earlier-diverging lineage. This aligns with the expectation that phylogenetically closer species exhibit more similar boundary features (Liu et al., 2018; Wu et al., 2020; Liu et al., 2022). This evolutionary framework effectively integrates and explains all findings from our morphological comparisons, genomic structural variations, and identification of molecular markers. This approach not only resolves taxonomic uncertainties but also establishes a solid phylogenetic foundation for subsequent conservation genetics studies targeting these endangered species.

### 4.4 Morphological stasis and cp. genome divergence

Our finding that morphologically similar *Madhuca* species harbor distinguishable cp. genome sequences is consistent with observations in other plant genera, e.g., *Oryza* (Liu et al., 2015), *Populus* (Schroeder et al., 2012; Schroeder and Fladung, 2015), and *Camellia* (Cai et al., 2025). This pattern reflects that non-coding regions of the plastome accumulate neutral or nearly neutral substitutions independently of morphological evolution.

Similar patterns of morphological stasis accompanied by genomic divergence have been observed in other plant lineages. In tree ferns (Cyatheaceae), for example, substantial genomic restructuring has occurred without pronounced morphological change (Wei et al., 2025). In plants, the functional interdependence between the nuclear and chloroplast genomes (Sloan et al., 2018) suggests that cytonuclear coevolution may contribute to such decoupling between phenotype and genotype (Forsythe et al., 2021). This framework could partly explain the phenotypic similarity yet plastid genome divergence observed among the three *Madhuca* species, although nuclear genomic data would be required to fully evaluate this hypothesis.

### 4.5 Implications for conservation and limitations of this study

The chloroplast genomic resources generated in this study offer a valuable foundation for the conservation of the endangered *M. pasquieri*. Although the SSRs and hypervariable regions were identified from cultivated individuals and thus remain to be validated at the population level, they provide a promising molecular approach for species discrimination—particularly when reproductive or juvenile materials render morphological diagnosis unreliable. Once validated, these molecular tools could be deployed to assess genetic diversity and population structure in the remaining wild populations, thereby guiding both *ex situ* and *in situ* conservation planning. Our phylogenetic reconstruction further highlights the evolutionary distinctiveness of *M. pasquieri*, reinforcing the need to treat it as a priority conservation unit.

Several limitations should be acknowledged. Because the phylogenetic inferences rely solely on the maternally inherited plastid genome, they capture a single evolutionary history and cannot resolve potential hybridization, introgression, or incomplete lineage sorting. The morphological comparisons are qualitative and lack quantitative morphometric support, which limits their resolution for species delimitation. Furthermore, as an endangered species, *M. pasquieri* is subject to restricted wild sampling access, and the present work is constrained to cultivated material. Despite these constraints, the molecular resources established here provide an actionable starting point. We are actively seeking to expand sampling to include additional wild populations, and future studies will integrate nuclear genomic data and quantitative morphological analyses to confirm the diagnostic utility of these molecular tools and to refine species boundaries.

## 5 Conclusion

This study provides molecular tools for the identification and elucidates phylogenetic relationships of endangered *M. pasquieri* through comparative cp. genome analysis with its congeners. The cp. genomes exhibited high structural and compositional conservation across the three *Madhuca* species. We identified hypervariable intergenic regions and SSRs that provide a molecular toolkit to aid species delimitation. Phylogenetic reconstruction strongly supported *M. pasquieri* and *M. hainanensis* as a sister clade, with *M. longifolia* as an earlier−diverging lineage. These findings offer preliminary phylogenetic insights for species delimitation and provide valuable genomic resources for the conservation of *M. pasquieri*.

## Data Availability

The raw sequence data for the cp. genomes of *M. pasquieri*, *M. hainanensis*, and *M. longifolia* presented in this study have been deposited in the GSA, National Genomics Data Center (NGDC), China National Center for Bioinformation BIG Sub-GSA (https://ngdc.cncb.ac.cn/gsa) under accession numbers CRA038892, BioProject ID: PRJCA058289.
