# Supplementary material for: Comparative analysis of morphology and chloroplast genomes in endangered plant Madhuca pasquieri and two congeneric plants: revealing phylogenetic relationships
Source: Front Plant Sci. 2026 Jun 19;17:1836586. doi: 10.3389/fpls.2026.1836586 (PMC13328432; doi:10.3389/fpls.2026.1836586)
Supplement: Supplementary file 1 [file Table1.docx]

**Table S1.** The codon usage of *Madhuca pasquieri.*

| No. | Amino Acid | Codon | Number | RSCU | Percentage/% |
| --- | --- | --- | --- | --- | --- |
| 1 | Arg | CGG | 82 | 0.39 | 5.98 |
|  |  | CGU | 290 | 1.39 |  |
|  |  | CGC | 71 | 0.34 |  |
|  |  | AGA | 403 | 1.94 |  |
|  |  | AGG | 124 | 0.6 |  |
|  |  | CGA | 279 | 1.34 |  |
|  | total |  | 1249 | 6 |  |
| 2 | Leu | UUA | 700 | 1.94 | 10.36 |
|  |  | CUG | 126 | 0.35 |  |
|  |  | CUU | 465 | 1.29 |  |
|  |  | CUC | 134 | 0.37 |  |
|  |  | UUG | 457 | 1.27 |  |
|  |  | CUA | 283 | 0.78 |  |
|  | total |  | 2165 | 6 |  |
| 3 | Ser | UCA | 309 | 1.18 | 7.51 |
|  |  | UCG | 132 | 0.5 |  |
|  |  | UCC | 247 | 0.94 |  |
|  |  | AGU | 328 | 1.25 |  |
|  |  | UCU | 471 | 1.8 |  |
|  |  | AGC | 82 | 0.31 |  |
|  | total |  | 1569 | 6 |  |
| 4 | Ala | GCC | 172 | 0.6 | 5.46 |
|  |  | GCU | 523 | 1.83 |  |
|  |  | GCG | 113 | 0.4 |  |
|  |  | GCA | 334 | 1.17 |  |
|  | total |  | 1142 | 4 |  |
| 5 | Gly | GGG | 252 | 0.7 | 6.87 |
|  |  | GGC | 153 | 0.43 |  |
|  |  | GGU | 466 | 1.3 |  |
|  |  | GGA | 564 | 1.57 |  |
|  | total |  | 1435 | 4 |  |
| 6 | Pro | CCU | 343 | 1.59 | 4.12 |
|  |  | CCC | 167 | 0.77 |  |
|  |  | CCG | 105 | 0.49 |  |
|  |  | CCA | 247 | 1.15 |  |
|  | total |  | 862 | 4 |  |
| 7 | Thr | ACA | 317 | 1.23 | 4.94 |
|  |  | ACU | 435 | 1.69 |  |
|  |  | ACC | 188 | 0.73 |  |
|  |  | ACG | 92 | 0.36 |  |
|  | total |  | 1032 | 4 |  |
| 8 | Val | GUG | 166 | 0.59 | 5.42 |
|  |  | GUC | 114 | 0.4 |  |
|  |  | GUU | 414 | 1.46 |  |
|  |  | GUA | 439 | 1.55 |  |
|  | total |  | 1133 | 4 |  |
| 9 | end | UGA | 14 | 0.81 | 0.25 |
|  |  | UAG | 9 | 0.52 |  |
|  |  | UAA | 29 | 1.67 |  |
|  | total |  | 52 | 3 |  |
| 10 | Ile | AUA | 573 | 0.95 | 8.70 |
|  |  | AUU | 892 | 1.47 |  |
|  |  | AUC | 353 | 0.58 |  |
|  | total |  | 1818 | 3 |  |
| 11 | Asn | AAC | 220 | 0.43 | 4.93 |
|  |  | AAU | 810 | 1.57 |  |
|  | total |  | 1030 | 2 |  |
| 12 | Asp | GAU | 706 | 1.63 | 4.14 |
|  |  | GAC | 160 | 0.37 |  |
|  | total |  | 866 | 2 |  |
| 13 | Cys | UGC | 55 | 0.49 | 1.08 |
|  |  | UGU | 170 | 1.51 |  |
|  | total |  | 225 | 2 |  |
| 14 | Gln | CAA | 584 | 1.51 | 3.69 |
|  |  | CAG | 187 | 0.49 |  |
|  | total |  | 771 | 2 |  |
| 15 | Glu | GAA | 863 | 1.55 | 5.34 |
|  |  | GAG | 252 | 0.45 |  |
|  | total |  | 1115 | 2 |  |
| 16 | His | CAC | 116 | 0.45 | 2.44 |
|  |  | CAU | 394 | 1.55 |  |
|  | total |  | 510 | 2 |  |
| 17 | Lys | AAG | 271 | 0.49 | 5.34 |
|  |  | AAA | 844 | 1.51 |  |
|  | total |  | 1115 | 2 |  |
| 18 | Phe | UUC | 390 | 0.68 | 5.50 |
|  |  | UUU | 759 | 1.32 |  |
|  | total |  | 1149 | 2 |  |
| 19 | Tyr | UAU | 644 | 1.61 | 3.82 |
|  |  | UAC | 154 | 0.39 |  |
|  | total |  | 798 | 2 |  |
| 20 | Met | AUG | 488 | 1 | 2.34 |
|  | total |  | 488 | 1 |  |
| 21 | Trp | UGG | 375 | 1 | 1.79 |
|  | total |  | 375 | 1 |  |

**Table S2.** The codon usage of *Madhuca hainanensis.*

| No. | Amino Acid | Codon | Number | RSCU | Percentage/% |
| --- | --- | --- | --- | --- | --- |
| 1 | Arg | AGA | 401 | 1.93 | 5.98 |
|  |  | CGA | 279 | 1.34 |  |
|  |  | CGU | 290 | 1.39 |  |
|  |  | CGG | 84 | 0.4 |  |
|  |  | AGG | 124 | 0.6 |  |
|  |  | CGC | 71 | 0.34 |  |
|  | total |  | 1249 | 6 |  |
| 2 | Leu | UUG | 454 | 1.26 | 10.35 |
|  |  | CUA | 283 | 0.78 |  |
|  |  | CUU | 464 | 1.29 |  |
|  |  | CUG | 125 | 0.35 |  |
|  |  | UUA | 704 | 1.95 |  |
|  |  | CUC | 134 | 0.37 |  |
|  | total |  | 2164 | 6 |  |
| 3 | Ser | UCC | 246 | 0.94 | 7.51 |
|  |  | UCG | 132 | 0.5 |  |
|  |  | AGU | 328 | 1.25 |  |
|  |  | UCU | 472 | 1.8 |  |
|  |  | AGC | 83 | 0.32 |  |
|  |  | UCA | 309 | 1.18 |  |
|  | total |  | 1570 | 6 |  |
| 4 | Ala | GCG | 113 | 0.4 | 5.46 |
|  |  | GCC | 172 | 0.6 |  |
|  |  | GCA | 334 | 1.17 |  |
|  |  | GCU | 523 | 1.83 |  |
|  | total |  | 1142 | 4 |  |
| 5 | Gly | GGC | 152 | 0.42 | 6.86 |
|  |  | GGG | 250 | 0.7 |  |
|  |  | GGU | 468 | 1.31 |  |
|  |  | GGA | 564 | 1.57 |  |
|  | total |  | 1434 | 4 |  |
| 6 | Pro | CCU | 343 | 1.59 | 4.12 |
|  |  | CCA | 247 | 1.15 |  |
|  |  | CCC | 167 | 0.77 |  |
|  |  | CCG | 105 | 0.49 |  |
|  | total |  | 862 | 4 |  |
| 7 | Thr | ACU | 436 | 1.69 | 4.94 |
|  |  | ACA | 317 | 1.23 |  |
|  |  | ACC | 187 | 0.72 |  |
|  |  | ACG | 92 | 0.36 |  |
|  | total |  | 1032 | 4 |  |
| 8 | Val | GUC | 114 | 0.4 | 5.42 |
|  |  | GUG | 165 | 0.58 |  |
|  |  | GUU | 414 | 1.46 |  |
|  |  | GUA | 440 | 1.55 |  |
|  | total |  | 1133 | 4 |  |
| 9 | end | UAG | 9 | 0.52 | 0.25 |
|  |  | UGA | 14 | 0.81 |  |
|  |  | UAA | 29 | 1.67 |  |
|  | total |  | 52 | 3 |  |
| 10 | Ile | AUA | 573 | 0.95 | 8.69 |
|  |  | AUU | 890 | 1.47 |  |
|  |  | AUC | 353 | 0.58 |  |
|  | total |  | 1816 | 3 |  |
| 11 | Asn | AAU | 809 | 1.57 | 4.92 |
|  |  | AAC | 220 | 0.43 |  |
|  | total |  | 1029 | 2 |  |
| 12 | Asp | GAC | 160 | 0.37 | 4.14 |
|  |  | GAU | 706 | 1.63 |  |
|  | total |  | 866 | 2 |  |
| 13 | Cys | UGC | 55 | 0.49 | 1.08 |
|  |  | UGU | 170 | 1.51 |  |
|  | total |  | 225 | 2 |  |
| 14 | Gln | CAA | 584 | 1.51 | 3.69 |
|  |  | CAG | 187 | 0.49 |  |
|  | total |  | 771 | 2 |  |
| 15 | Glu | GAG | 252 | 0.45 | 5.34 |
|  |  | GAA | 863 | 1.55 |  |
|  | total |  | 1115 | 2 |  |
| 16 | His | CAU | 395 | 1.55 | 2.45 |
|  |  | CAC | 116 | 0.45 |  |
|  | total |  | 511 | 2 |  |
| 17 | Lys | AAA | 842 | 1.51 | 5.34 |
|  |  | AAG | 273 | 0.49 |  |
|  | total |  | 1115 | 2 |  |
| 18 | Phe | UUC | 389 | 0.68 | 5.51 |
|  |  | UUU | 762 | 1.32 |  |
|  | total |  | 1151 | 2 |  |
| 19 | Tyr | UAC | 154 | 0.39 | 3.82 |
|  |  | UAU | 644 | 1.61 |  |
|  | total |  | 798 | 2 |  |
| 20 | Met | AUG | 489 | 1 | 2.34 |
|  | total |  | 489 | 1 |  |
| 21 | Trp | UGG | 375 | 1 | 1.79 |
|  | total |  | 375 | 1 |  |

**Table S3.** The codon usage of *Madhuca longifolia*

| No. | Amino Acid | Codon | Number | RSCU | Percentage/% |
| --- | --- | --- | --- | --- | --- |
| 1 | Arg | CGG | 83 | 0.4 | 5.97 |
|  |  | CGA | 279 | 1.34 |  |
|  |  | AGA | 403 | 1.94 |  |
|  |  | AGG | 123 | 0.59 |  |
|  |  | CGC | 71 | 0.34 |  |
|  |  | CGU | 289 | 1.39 |  |
|  | total |  | 1248 | 6 |  |
| 2 | Leu | CUG | 128 | 0.35 | 10.36 |
|  |  | CUA | 284 | 0.79 |  |
|  |  | CUC | 134 | 0.37 |  |
|  |  | UUA | 699 | 1.94 |  |
|  |  | UUG | 458 | 1.27 |  |
|  |  | CUU | 462 | 1.28 |  |
|  | total |  | 2165 | 6 |  |
| 3 | Ser | AGC | 85 | 0.32 | 7.53 |
|  |  | UCU | 473 | 1.8 |  |
|  |  | AGU | 327 | 1.25 |  |
|  |  | UCA | 311 | 1.19 |  |
|  |  | UCG | 133 | 0.51 |  |
|  |  | UCC | 244 | 0.93 |  |
|  | total |  | 1573 | 6 |  |
| 4 | Ala | GCU | 520 | 1.83 | 5.45 |
|  |  | GCC | 171 | 0.6 |  |
|  |  | GCA | 336 | 1.18 |  |
|  |  | GCG | 112 | 0.39 |  |
|  | total |  | 1139 | 4 |  |
| 5 | Gly | GGU | 469 | 1.31 | 6.87 |
|  |  | GGC | 151 | 0.42 |  |
|  |  | GGA | 565 | 1.57 |  |
|  |  | GGG | 250 | 0.7 |  |
|  | total |  | 1435 | 4 |  |
| 6 | Pro | CCG | 104 | 0.48 | 4.12 |
|  |  | CCA | 247 | 1.15 |  |
|  |  | CCC | 167 | 0.77 |  |
|  |  | CCU | 344 | 1.6 |  |
|  | total |  | 862 | 4 |  |
| 7 | Thr | ACC | 191 | 0.74 | 4.96 |
|  |  | ACA | 317 | 1.22 |  |
|  |  | ACG | 93 | 0.36 |  |
|  |  | ACU | 435 | 1.68 |  |
|  | total |  | 1036 | 4 |  |
| 8 | Val | GUU | 414 | 1.46 | 5.42 |
|  |  | GUC | 114 | 0.41 |  |
|  |  | GUA | 442 | 1.56 |  |
|  |  | GUG | 162 | 0.57 |  |
|  | total |  | 1132 | 4 |  |
| 9 | end | UGA | 14 | 0.81 | 0.25 |
|  |  | UAA | 29 | 1.67 |  |
|  |  | UAG | 9 | 0.52 |  |
|  | total |  | 52 | 3 |  |
| 10 | Ile | AUC | 353 | 0.58 | 8.69 |
|  |  | AUA | 572 | 0.94 |  |
|  |  | AUU | 892 | 1.47 |  |
|  | total |  | 1817 | 3 |  |
| 11 | Asn | AAC | 219 | 0.43 | 4.92 |
|  |  | AAU | 809 | 1.57 |  |
|  | total |  | 1028 | 2 |  |
| 12 | Asp | GAU | 705 | 1.63 | 4.13 |
|  |  | GAC | 159 | 0.37 |  |
|  | total |  | 864 | 2 |  |
| 13 | Cys | UGU | 170 | 1.51 | 1.08 |
|  |  | UGC | 55 | 0.49 |  |
|  | total |  | 225 | 2 |  |
| 14 | Gln | CAG | 187 | 0.48 | 3.69 |
|  |  | CAA | 585 | 1.52 |  |
|  | total |  | 772 | 2 |  |
| 15 | Glu | GAA | 863 | 1.55 | 5.34 |
|  |  | GAG | 252 | 0.45 |  |
|  | total |  | 1115 | 2 |  |
| 16 | His | CAC | 117 | 0.46 | 2.45 |
|  |  | CAU | 394 | 1.54 |  |
|  | total |  | 511 | 2 |  |
| 17 | Lys | AAA | 843 | 1.51 | 5.33 |
|  |  | AAG | 271 | 0.49 |  |
|  | total |  | 1114 | 2 |  |
| 18 | Phe | UUU | 766 | 1.33 | 5.52 |
|  |  | UUC | 387 | 0.67 |  |
|  | total |  | 1153 | 2 |  |
| 19 | Tyr | UAU | 643 | 1.61 | 3.82 |
|  |  | UAC | 155 | 0.39 |  |
|  | total |  | 798 | 2 |  |
| 20 | Met | AUG | 486 | 1 | 2.33 |
|  | total |  | 486 | 1 |  |
| 21 | Trp | UGG | 374 | 1 | 1.79 |
|  | total |  | 374 | 1 |  |

**Table S4** Tandem repeat sequences in the chloroplast genome of *M. pasquieri.*

| **Indices** | **Period Size** | **Copy Number** | **Consensus Size** | **Percent Matches** | **Percent Indels** | **Score** | **A** | **C** | **G** | **T** | **Entropy (0-2)** |
| --- | --- | --- | --- | --- | --- | --- | --- | --- | --- | --- | --- |
| 419-511 | 3 | 32.7 | 3 | 73 | 10 | 79 | 61 | 2 | 2 | 34 | 1.2 |
| 432-511 | 20 | 4.3 | 18 | 84 | 9 | 83 | 61 | 1 | 2 | 35 | 1.18 |
| 424-508 | 17 | 4.8 | 18 | 79 | 13 | 86 | 60 | 2 | 2 | 35 | 1.23 |
| 3939-3966 | 1 | 28 | 1 | 100 | 0 | 56 | 0 | 0 | 0 | 100 | 0 |
| 4507-4571 | 27 | 2.4 | 27 | 94 | 0 | 112 | 53 | 6 | 16 | 23 | 1.65 |
| 5110-5157 | 11 | 4.2 | 11 | 75 | 19 | 53 | 20 | 2 | 0 | 77 | 0.88 |
| 6740-6790 | 23 | 2.2 | 23 | 83 | 16 | 70 | 70 | 0 | 3 | 25 | 1.04 |
| 6755-6803 | 11 | 4.3 | 11 | 79 | 7 | 53 | 73 | 0 | 2 | 24 | 0.94 |
| 6776-6851 | 11 | 6.8 | 11 | 73 | 16 | 59 | 67 | 1 | 0 | 31 | 0.99 |
| 6784-6871 | 12 | 7.4 | 12 | 75 | 20 | 87 | 63 | 1 | 0 | 35 | 1.02 |
| 6780-6875 | 50 | 2 | 48 | 86 | 12 | 133 | 64 | 1 | 0 | 34 | 1.01 |
| 6760-6897 | 45 | 2.9 | 45 | 76 | 18 | 122 | 64 | 1 | 0 | 33 | 1.08 |
| 6832-6913 | 28 | 3 | 27 | 78 | 8 | 76 | 67 | 1 | 1 | 30 | 1.06 |
| 9365-9399 | 17 | 2 | 18 | 94 | 5 | 63 | 31 | 5 | 5 | 57 | 1.46 |
| 10270-10297 | 12 | 2.3 | 12 | 100 | 0 | 56 | 64 | 7 | 14 | 14 | 1.48 |
| 11444-11491 | 15 | 3.8 | 13 | 77 | 22 | 57 | 31 | 12 | 0 | 56 | 1.37 |
| 11463-11491 | 15 | 1.9 | 15 | 100 | 0 | 58 | 27 | 17 | 0 | 55 | 1.42 |
| 13353-13378 | 1 | 26 | 1 | 100 | 0 | 52 | 100 | 0 | 0 | 0 | 0 |
| 14765-14789 | 12 | 2.1 | 12 | 100 | 0 | 50 | 56 | 8 | 0 | 36 | 1.29 |
| 31157-31182 | 12 | 2.2 | 12 | 100 | 0 | 52 | 7 | 0 | 7 | 84 | 0.77 |
| 31525-31560 | 18 | 2 | 18 | 100 | 0 | 72 | 55 | 0 | 0 | 44 | 0.99 |
| 31517-31541 | 12 | 2.1 | 12 | 100 | 0 | 50 | 44 | 0 | 0 | 56 | 0.99 |
| 31725-31779 | 29 | 1.9 | 28 | 92 | 3 | 92 | 40 | 9 | 9 | 41 | 1.68 |
| 33932-33985 | 17 | 3.2 | 17 | 86 | 5 | 74 | 44 | 1 | 5 | 48 | 1.37 |
| 33916-33997 | 38 | 2.2 | 38 | 84 | 4 | 103 | 42 | 1 | 6 | 50 | 1.35 |
| 35355-35404 | 17 | 2.9 | 17 | 77 | 14 | 57 | 32 | 8 | 0 | 60 | 1.26 |
| 35746-35779 | 17 | 2 | 17 | 88 | 11 | 52 | 82 | 0 | 8 | 8 | 0.85 |
| 46775-46802 | 14 | 2 | 14 | 100 | 0 | 56 | 28 | 14 | 7 | 50 | 1.69 |
| 50016-50040 | 12 | 2.1 | 12 | 100 | 0 | 50 | 44 | 8 | 16 | 32 | 1.76 |
| 50429-50478 | 22 | 2.3 | 22 | 93 | 3 | 84 | 44 | 0 | 6 | 50 | 1.26 |
| 50448-50487 | 18 | 2.2 | 18 | 95 | 0 | 71 | 45 | 0 | 7 | 47 | 1.31 |
| 50431-50506 | 22 | 3.2 | 22 | 77 | 14 | 64 | 44 | 0 | 7 | 47 | 1.32 |
| 50800-50858 | 26 | 2.4 | 24 | 83 | 10 | 75 | 40 | 8 | 0 | 50 | 1.33 |
| 50800-50859 | 12 | 4.9 | 12 | 82 | 13 | 70 | 40 | 8 | 0 | 51 | 1.32 |
| 50832-50865 | 17 | 2 | 17 | 94 | 0 | 59 | 38 | 8 | 0 | 52 | 1.33 |
| 55102-55152 | 19 | 2.5 | 21 | 78 | 9 | 61 | 25 | 3 | 1 | 68 | 1.17 |
| 62604-62643 | 16 | 2.5 | 16 | 91 | 0 | 62 | 32 | 12 | 7 | 47 | 1.69 |
| 63071-63102 | 16 | 2 | 16 | 100 | 0 | 64 | 43 | 6 | 0 | 50 | 1.27 |
| 63195-63247 | 14 | 3.8 | 14 | 80 | 14 | 65 | 35 | 9 | 7 | 47 | 1.64 |
| 63189-63247 | 21 | 2.9 | 21 | 81 | 18 | 72 | 37 | 8 | 8 | 45 | 1.65 |
| 63553-63593 | 20 | 2 | 20 | 100 | 0 | 82 | 41 | 9 | 19 | 29 | 1.83 |
| 64884-64915 | 12 | 2.7 | 12 | 95 | 0 | 55 | 15 | 6 | 0 | 78 | 0.95 |
| 67563-67604 | 11 | 3.8 | 11 | 80 | 0 | 57 | 59 | 9 | 0 | 30 | 1.29 |
| 67562-67610 | 22 | 2.2 | 22 | 96 | 0 | 89 | 57 | 10 | 0 | 32 | 1.32 |
| 67554-67613 | 22 | 3.2 | 17 | 75 | 22 | 57 | 61 | 8 | 0 | 30 | 1.25 |
| 75715-75747 | 16 | 2.1 | 16 | 100 | 0 | 66 | 48 | 15 | 0 | 36 | 1.45 |
| 93603-93657 | 24 | 2.5 | 22 | 85 | 8 | 67 | 14 | 23 | 12 | 49 | 1.78 |
| 93625-93692 | 21 | 3.2 | 21 | 77 | 8 | 75 | 11 | 22 | 8 | 57 | 1.61 |
| 96061-96116 | 18 | 3.1 | 18 | 97 | 0 | 103 | 30 | 8 | 26 | 33 | 1.87 |
| 101730-101771 | 21 | 2 | 21 | 95 | 0 | 75 | 19 | 21 | 16 | 42 | 1.89 |
| 104047-104084 | 18 | 2.1 | 18 | 90 | 0 | 58 | 23 | 7 | 7 | 60 | 1.51 |
| 112224-112288 | 32 | 2 | 32 | 96 | 0 | 121 | 41 | 23 | 9 | 26 | 1.84 |
| 116578-116619 | 20 | 2 | 21 | 86 | 13 | 61 | 50 | 26 | 0 | 23 | 1.5 |
| 117840-117879 | 20 | 2.1 | 19 | 85 | 4 | 53 | 37 | 2 | 7 | 52 | 1.43 |
| 118839-118875 | 18 | 2.1 | 18 | 100 | 0 | 74 | 32 | 16 | 0 | 51 | 1.45 |
| 128778-128811 | 17 | 2 | 17 | 100 | 0 | 68 | 41 | 11 | 11 | 35 | 1.78 |
| 136154-136218 | 32 | 2 | 32 | 96 | 0 | 121 | 26 | 9 | 23 | 41 | 1.84 |
| 144358-144395 | 18 | 2.1 | 18 | 90 | 0 | 58 | 60 | 7 | 7 | 23 | 1.51 |
| 146671-146712 | 21 | 2 | 21 | 95 | 0 | 75 | 42 | 16 | 21 | 19 | 1.89 |
| 152326-152381 | 18 | 3.1 | 18 | 97 | 0 | 103 | 33 | 26 | 8 | 30 | 1.87 |
| 154750-154839 | 21 | 4.1 | 22 | 75 | 8 | 76 | 54 | 10 | 23 | 12 | 1.67 |

**Table S5** Tandem repeat sequences in the chloroplast genome of *M. hainanensis.*

| **Indices** | **Period Size** | **Copy Number** | **Consensus Size** | **Percent Matches** | **Percent Indels** | **Score** | **A** | **C** | **G** | **T** | **Entropy (0-2)** |
| --- | --- | --- | --- | --- | --- | --- | --- | --- | --- | --- | --- |
| 246-281 | 18 | 1.9 | 18 | 88 | 5 | 54 | 50 | 5 | 13 | 30 | 1.65 |
| 418-510 | 3 | 32.7 | 3 | 73 | 10 | 79 | 61 | 2 | 2 | 34 | 1.2 |
| 431-510 | 20 | 4.3 | 18 | 84 | 9 | 83 | 61 | 1 | 2 | 35 | 1.18 |
| 423-507 | 17 | 4.8 | 18 | 79 | 13 | 86 | 60 | 2 | 2 | 35 | 1.23 |
| 4497-4561 | 27 | 2.4 | 27 | 94 | 0 | 112 | 53 | 6 | 16 | 23 | 1.65 |
| 5098-5145 | 11 | 4.2 | 11 | 75 | 19 | 53 | 20 | 2 | 0 | 77 | 0.88 |
| 6728-6778 | 23 | 2.2 | 23 | 83 | 16 | 70 | 70 | 0 | 3 | 25 | 1.04 |
| 6743-6791 | 11 | 4.3 | 11 | 79 | 7 | 53 | 73 | 0 | 2 | 24 | 0.94 |
| 6764-6839 | 11 | 6.8 | 11 | 73 | 16 | 59 | 67 | 1 | 0 | 31 | 0.99 |
| 6772-6859 | 12 | 7.4 | 12 | 75 | 20 | 87 | 63 | 1 | 0 | 35 | 1.02 |
| 6768-6863 | 50 | 2 | 48 | 86 | 12 | 133 | 64 | 1 | 0 | 34 | 1.01 |
| 6748-6885 | 45 | 2.9 | 45 | 76 | 18 | 122 | 64 | 1 | 0 | 33 | 1.08 |
| 6820-6901 | 28 | 3 | 27 | 78 | 8 | 76 | 67 | 1 | 1 | 30 | 1.06 |
| 9360-9394 | 17 | 2 | 18 | 94 | 5 | 63 | 31 | 5 | 5 | 57 | 1.46 |
| 10264-10291 | 12 | 2.3 | 12 | 100 | 0 | 56 | 64 | 7 | 14 | 14 | 1.48 |
| 11435-11482 | 15 | 3.8 | 13 | 77 | 22 | 57 | 31 | 12 | 0 | 56 | 1.37 |
| 11454-11482 | 15 | 1.9 | 15 | 100 | 0 | 58 | 27 | 17 | 0 | 55 | 1.42 |
| 14753-14777 | 12 | 2.1 | 12 | 100 | 0 | 50 | 56 | 8 | 0 | 36 | 1.29 |
| 31146-31171 | 12 | 2.2 | 12 | 100 | 0 | 52 | 7 | 0 | 7 | 84 | 0.77 |
| 31514-31567 | 18 | 3 | 18 | 100 | 0 | 108 | 55 | 0 | 0 | 44 | 0.99 |
| 31506-31530 | 12 | 2.1 | 12 | 100 | 0 | 50 | 44 | 0 | 0 | 56 | 0.99 |
| 31732-31786 | 29 | 1.9 | 28 | 92 | 3 | 92 | 40 | 9 | 9 | 41 | 1.68 |
| 33938-33991 | 17 | 3.2 | 17 | 86 | 5 | 74 | 44 | 1 | 5 | 48 | 1.37 |
| 33922-34003 | 38 | 2.2 | 38 | 84 | 4 | 103 | 42 | 1 | 6 | 50 | 1.35 |
| 35358-35407 | 17 | 2.9 | 17 | 77 | 14 | 57 | 32 | 8 | 0 | 60 | 1.26 |
| 35749-35783 | 18 | 1.9 | 18 | 88 | 11 | 54 | 82 | 0 | 8 | 8 | 0.83 |
| 46782-46809 | 14 | 2 | 14 | 100 | 0 | 56 | 28 | 14 | 7 | 50 | 1.69 |
| 50026-50050 | 12 | 2.1 | 12 | 100 | 0 | 50 | 44 | 8 | 16 | 32 | 1.76 |
| 50439-50488 | 22 | 2.3 | 22 | 93 | 3 | 84 | 44 | 0 | 6 | 50 | 1.26 |
| 50458-50497 | 18 | 2.2 | 18 | 95 | 0 | 71 | 45 | 0 | 7 | 47 | 1.31 |
| 50441-50516 | 22 | 3.2 | 22 | 77 | 14 | 64 | 44 | 0 | 7 | 47 | 1.32 |
| 50810-50868 | 26 | 2.4 | 24 | 83 | 10 | 75 | 40 | 8 | 0 | 50 | 1.33 |
| 50810-50869 | 12 | 4.9 | 12 | 82 | 13 | 70 | 40 | 8 | 0 | 51 | 1.32 |
| 50842-50875 | 17 | 2 | 17 | 94 | 0 | 59 | 38 | 8 | 0 | 52 | 1.33 |
| 55114-55164 | 19 | 2.5 | 21 | 78 | 9 | 61 | 25 | 3 | 1 | 68 | 1.17 |
| 63067-63098 | 16 | 2 | 16 | 100 | 0 | 64 | 43 | 6 | 0 | 50 | 1.27 |
| 63104-63151 | 22 | 2.2 | 21 | 96 | 3 | 87 | 54 | 4 | 8 | 33 | 1.5 |
| 63213-63265 | 14 | 3.8 | 14 | 85 | 14 | 74 | 35 | 9 | 9 | 45 | 1.69 |
| 63472-63499 | 1 | 28 | 1 | 100 | 0 | 56 | 0 | 0 | 0 | 100 | 0 |
| 63582-63622 | 20 | 2 | 20 | 100 | 0 | 82 | 41 | 9 | 19 | 29 | 1.83 |
| 64913-64944 | 12 | 2.7 | 12 | 95 | 0 | 55 | 15 | 6 | 0 | 78 | 0.95 |
| 67593-67656 | 11 | 5.8 | 11 | 77 | 0 | 74 | 59 | 9 | 0 | 31 | 1.29 |
| 67606-67662 | 6 | 10.2 | 6 | 74 | 14 | 50 | 57 | 10 | 0 | 31 | 1.32 |
| 67592-67662 | 22 | 3.2 | 22 | 97 | 0 | 133 | 57 | 9 | 0 | 32 | 1.31 |
| 67634-67665 | 17 | 1.9 | 17 | 93 | 6 | 57 | 62 | 9 | 0 | 28 | 1.26 |
| 75762-75794 | 16 | 2.1 | 16 | 100 | 0 | 66 | 48 | 15 | 0 | 36 | 1.45 |
| 93651-93705 | 24 | 2.5 | 22 | 85 | 8 | 67 | 14 | 23 | 12 | 49 | 1.78 |
| 93673-93740 | 21 | 3.2 | 21 | 77 | 8 | 75 | 11 | 22 | 8 | 57 | 1.61 |
| 96109-96164 | 18 | 3.1 | 18 | 97 | 0 | 103 | 30 | 8 | 26 | 33 | 1.87 |
| 101778-101819 | 21 | 2 | 21 | 95 | 0 | 75 | 19 | 21 | 16 | 42 | 1.89 |
| 104095-104132 | 18 | 2.1 | 18 | 90 | 0 | 58 | 23 | 7 | 7 | 60 | 1.51 |
| 112271-112335 | 32 | 2 | 32 | 96 | 0 | 121 | 41 | 23 | 9 | 26 | 1.84 |
| 116625-116666 | 20 | 2 | 21 | 86 | 13 | 61 | 50 | 26 | 0 | 23 | 1.5 |
| 117442-117470 | 14 | 2.1 | 14 | 100 | 0 | 58 | 44 | 13 | 6 | 34 | 1.71 |
| 117886-117925 | 20 | 2.1 | 19 | 85 | 4 | 53 | 37 | 2 | 7 | 52 | 1.43 |
| 118888-118924 | 18 | 2.1 | 18 | 100 | 0 | 74 | 32 | 16 | 0 | 51 | 1.45 |
| 128830-128863 | 17 | 2 | 17 | 100 | 0 | 68 | 41 | 11 | 11 | 35 | 1.78 |
| 136206-136270 | 32 | 2 | 32 | 96 | 0 | 121 | 26 | 9 | 23 | 41 | 1.84 |
| 144409-144446 | 18 | 2.1 | 18 | 90 | 0 | 58 | 60 | 7 | 7 | 23 | 1.51 |
| 146722-146763 | 21 | 2 | 21 | 95 | 0 | 75 | 42 | 16 | 21 | 19 | 1.89 |
| 152377-152432 | 18 | 3.1 | 18 | 97 | 0 | 103 | 33 | 26 | 8 | 30 | 1.87 |
| 154801-154890 | 21 | 4.1 | 22 | 75 | 8 | 76 | 54 | 10 | 23 | 12 | 1.67 |

**Table S6** Tandem repeat sequences in the chloroplast genome of *M. longifolia.*

| **Indices** | **Period Size** | **Copy Number** | **Consensus Size** | **Percent Matches** | **Percent Indels** | **Score** | **A** | **C** | **G** | **T** | **Entropy (0-2)** |
| --- | --- | --- | --- | --- | --- | --- | --- | --- | --- | --- | --- |
| 244-279 | 18 | 1.9 | 18 | 88 | 5 | 54 | 50 | 5 | 13 | 30 | 1.65 |
| 416-512 | 3 | 33.7 | 3 | 73 | 12 | 78 | 61 | 2 | 2 | 34 | 1.19 |
| 429-512 | 20 | 4.3 | 19 | 81 | 15 | 84 | 61 | 1 | 2 | 34 | 1.16 |
| 3941-3965 | 1 | 25 | 1 | 100 | 0 | 50 | 0 | 0 | 0 | 100 | 0 |
| 4511-4575 | 27 | 2.4 | 27 | 94 | 0 | 112 | 53 | 6 | 16 | 23 | 1.65 |
| 6659-6689 | 16 | 1.9 | 16 | 100 | 0 | 62 | 38 | 0 | 0 | 61 | 0.96 |
| 6653-6702 | 16 | 2.7 | 19 | 76 | 23 | 61 | 40 | 2 | 2 | 56 | 1.22 |
| 6694-6736 | 21 | 2 | 21 | 100 | 0 | 86 | 23 | 4 | 0 | 72 | 1.04 |
| 6753-6803 | 23 | 2.2 | 23 | 83 | 16 | 70 | 70 | 0 | 3 | 25 | 1.04 |
| 6768-6816 | 11 | 4.3 | 11 | 79 | 7 | 53 | 73 | 0 | 2 | 24 | 0.94 |
| 6789-6864 | 11 | 6.8 | 11 | 70 | 16 | 50 | 65 | 1 | 0 | 32 | 1.01 |
| 6793-6888 | 50 | 2 | 48 | 84 | 12 | 124 | 63 | 1 | 0 | 35 | 1.01 |
| 6845-6926 | 28 | 3 | 27 | 78 | 8 | 76 | 65 | 2 | 1 | 30 | 1.13 |
| 9379-9413 | 17 | 2 | 18 | 94 | 5 | 63 | 31 | 5 | 5 | 57 | 1.46 |
| 10290-10317 | 12 | 2.3 | 12 | 100 | 0 | 56 | 64 | 7 | 14 | 14 | 1.48 |
| 11171-11195 | 13 | 1.9 | 13 | 100 | 0 | 50 | 8 | 0 | 0 | 92 | 0.4 |
| 11463-11510 | 15 | 3.8 | 13 | 77 | 22 | 57 | 31 | 12 | 0 | 56 | 1.37 |
| 11482-11510 | 15 | 1.9 | 15 | 100 | 0 | 58 | 27 | 17 | 0 | 55 | 1.42 |
| 14790-14814 | 12 | 2.1 | 12 | 100 | 0 | 50 | 56 | 8 | 0 | 36 | 1.29 |
| 30524-30563 | 20 | 2 | 20 | 95 | 0 | 71 | 27 | 17 | 5 | 50 | 1.67 |
| 31216-31241 | 12 | 2.2 | 12 | 100 | 0 | 52 | 7 | 0 | 7 | 84 | 0.77 |
| 31584-31619 | 18 | 2 | 18 | 100 | 0 | 72 | 55 | 0 | 0 | 44 | 0.99 |
| 31576-31600 | 12 | 2.1 | 12 | 100 | 0 | 50 | 44 | 0 | 0 | 56 | 0.99 |
| 31784-31838 | 29 | 1.9 | 28 | 92 | 3 | 92 | 38 | 9 | 9 | 43 | 1.68 |
| 33991-34044 | 17 | 3.2 | 17 | 86 | 5 | 74 | 44 | 1 | 5 | 48 | 1.37 |
| 33975-34056 | 38 | 2.2 | 38 | 84 | 4 | 103 | 42 | 1 | 6 | 50 | 1.35 |
| 35411-35465 | 17 | 3.2 | 17 | 80 | 12 | 67 | 30 | 7 | 0 | 61 | 1.23 |
| 35434-35485 | 25 | 2.1 | 25 | 100 | 0 | 104 | 32 | 11 | 0 | 55 | 1.36 |
| 35827-35867 | 13 | 3.4 | 12 | 80 | 19 | 50 | 82 | 0 | 9 | 7 | 0.83 |
| 46867-46894 | 14 | 2 | 14 | 100 | 0 | 56 | 28 | 14 | 7 | 50 | 1.69 |
| 49073-49098 | 13 | 2 | 13 | 100 | 0 | 52 | 7 | 23 | 38 | 30 | 1.83 |
| 50114-50139 | 13 | 2 | 13 | 100 | 0 | 52 | 46 | 7 | 15 | 30 | 1.74 |
| 50523-50586 | 22 | 2.9 | 22 | 88 | 6 | 94 | 43 | 0 | 6 | 50 | 1.27 |
| 50899-50957 | 26 | 2.4 | 24 | 83 | 10 | 75 | 40 | 8 | 0 | 50 | 1.33 |
| 50899-50958 | 12 | 4.9 | 12 | 82 | 13 | 70 | 40 | 8 | 0 | 51 | 1.32 |
| 50931-50964 | 17 | 2 | 17 | 94 | 0 | 59 | 38 | 8 | 0 | 52 | 1.33 |
| 62717-62756 | 16 | 2.5 | 16 | 91 | 0 | 62 | 32 | 12 | 7 | 47 | 1.69 |
| 63184-63215 | 16 | 2 | 16 | 100 | 0 | 64 | 43 | 6 | 0 | 50 | 1.27 |
| 63221-63268 | 22 | 2.2 | 21 | 96 | 3 | 87 | 54 | 4 | 8 | 33 | 1.5 |
| 63324-63382 | 21 | 2.9 | 21 | 80 | 14 | 63 | 38 | 6 | 8 | 45 | 1.61 |
| 63330-63375 | 13 | 3.4 | 14 | 82 | 11 | 51 | 39 | 6 | 8 | 45 | 1.61 |
| 63589-63614 | 1 | 26 | 1 | 100 | 0 | 52 | 0 | 0 | 0 | 100 | 0 |
| 65030-65055 | 12 | 2.2 | 12 | 100 | 0 | 52 | 15 | 7 | 0 | 76 | 0.99 |
| 67691-67728 | 17 | 2.2 | 18 | 90 | 4 | 60 | 63 | 7 | 0 | 28 | 1.23 |
| 75825-75857 | 16 | 2.1 | 16 | 100 | 0 | 66 | 48 | 15 | 0 | 36 | 1.45 |
| 93734-93788 | 24 | 2.5 | 22 | 85 | 8 | 67 | 14 | 23 | 12 | 49 | 1.78 |
| 93756-93823 | 21 | 3.2 | 21 | 77 | 8 | 75 | 11 | 22 | 8 | 57 | 1.61 |
| 96192-96247 | 18 | 3.1 | 18 | 97 | 0 | 103 | 30 | 8 | 26 | 33 | 1.87 |
| 101861-101902 | 21 | 2 | 21 | 95 | 0 | 75 | 19 | 21 | 16 | 42 | 1.89 |
| 104178-104215 | 18 | 2.1 | 18 | 90 | 0 | 58 | 23 | 7 | 7 | 60 | 1.51 |
| 112347-112411 | 32 | 2 | 32 | 96 | 0 | 121 | 41 | 23 | 9 | 26 | 1.84 |
| 116701-116742 | 20 | 2 | 21 | 86 | 13 | 61 | 50 | 26 | 0 | 23 | 1.5 |
| 117501-117529 | 14 | 2.1 | 14 | 100 | 0 | 58 | 44 | 13 | 6 | 34 | 1.71 |
| 117544-117593 | 24 | 2.1 | 24 | 100 | 0 | 100 | 42 | 0 | 22 | 36 | 1.54 |
| 117972-118011 | 20 | 2.1 | 19 | 85 | 4 | 53 | 37 | 2 | 7 | 52 | 1.43 |
| 118966-119002 | 18 | 2.1 | 18 | 100 | 0 | 74 | 32 | 16 | 0 | 51 | 1.45 |
| 128898-128931 | 17 | 2 | 17 | 100 | 0 | 68 | 41 | 11 | 11 | 35 | 1.78 |
| 136274-136338 | 32 | 2 | 32 | 96 | 0 | 121 | 26 | 9 | 23 | 41 | 1.84 |
| 144470-144507 | 18 | 2.1 | 18 | 90 | 0 | 58 | 60 | 7 | 7 | 23 | 1.51 |
| 146783-146824 | 21 | 2 | 21 | 95 | 0 | 75 | 42 | 16 | 21 | 19 | 1.89 |
| 152438-152493 | 18 | 3.1 | 18 | 97 | 0 | 103 | 33 | 26 | 8 | 30 | 1.87 |
| 154862-154951 | 21 | 4.1 | 22 | 75 | 8 | 76 | 54 | 10 | 23 | 12 | 1.67 |

**Table S7.** Databases of comparative genome analysis species.

| **Species** | **Database** |
| --- | --- |
| *Sideroxylon wightianum* | https://www.ncbi.nlm.nih.gov/nuccore/MG719834.1/ |
| *Manilkara zapota* | https://www.ncbi.nlm.nih.gov/nuccore/MN295595.1/ |
| *Mimusops elengi* | https://www.ncbi.nlm.nih.gov/nuccore/NC_061660 |
| *Synsepalum dulcificum* | https://www.ncbi.nlm.nih.gov/nuccore/NC_053849 |
| *Gambeya africana* | https://www.ncbi.nlm.nih.gov/nuccore/MZ274141 |
| *Gambeya lacourtiana* | https://www.ncbi.nlm.nih.gov/nuccore/MZ274138 |
| *Gambeya gigantea* | https://www.ncbi.nlm.nih.gov/nuccore/MZ274140 |
| *Chrysophyllum cainito* | https://www.ncbi.nlm.nih.gov/nuccore/MT435527 |
| *Pouteria viridis* | https://www.ncbi.nlm.nih.gov/nuccore/OP650217 |
| *Pouteria campechiana* | https://www.ncbi.nlm.nih.gov/nuccore/KX426215 |
| *Pouteria caimito* | https://www.ncbi.nlm.nih.gov/nuccore/MN065160 |
| *Amborella trichopoda* | https://www.ncbi.nlm.nih.gov/nuccore/NC_005086.1 |
